# Creatinine muscle index in UK Biobank: comparison with MRI and associations with frailty and mortality

**DOI:** 10.1093/ckj/sfag139

**Published:** 2026-05-09

**Authors:** Giada Azzopardi, Thomas Davies, Myles J Lewis, Zudin Puthucheary, John R Prowle

**Affiliations:** Adult Critical Care Unit, The Royal London Hospital, Barts Health NHS Trust, London, UK; Critical Care and Peri-operative Medicine Research Group, William Harvey Research Institute, Barts and the London School of Medicine and Dentistry, Queen Mary University of London, London, UK; Critical Care and Peri-operative Medicine Research Group, William Harvey Research Institute, Barts and the London School of Medicine and Dentistry, Queen Mary University of London, London, UK; Centre for Centre for Experimental Medicine and Rheumatology, William Harvey Research Institute, Barts and the London School of Medicine and Dentistry, Queen Mary University of London, London, UK; Adult Critical Care Unit, The Royal London Hospital, Barts Health NHS Trust, London, UK; Critical Care and Peri-operative Medicine Research Group, William Harvey Research Institute, Barts and the London School of Medicine and Dentistry, Queen Mary University of London, London, UK; Adult Critical Care Unit, The Royal London Hospital, Barts Health NHS Trust, London, UK; Critical Care and Peri-operative Medicine Research Group, William Harvey Research Institute, Barts and the London School of Medicine and Dentistry, Queen Mary University of London, London, UK

**Keywords:** biomarker, frailty, multimorbidity, sarcopenia

## Abstract

**Background:**

Skeletal muscle wasting due to aging, acute illness, and chronic disease is associated with adverse outcomes including mortality, frailty, and multi-morbidity. We investigated the relationship of creatinine muscle index (CMI), a serum biological signature of muscle mass, in UK Biobank participants with gold standard muscle measurement and adverse outcomes.

**Method:**

We compared CMI with Magnetic Resonance Imaging (MRI) measured muscle mass in 33 799 participants using linear regression. We then assessed CMI’s ability to discriminate low muscle mass (≤2.5 SD below the mean). In the full UK Biobank cohort (*n* = 450 812), we utilized Cox-proportional hazards models to investigate associations between CMI with mortality, frailty and comorbidity.

**Results:**

CMI demonstrated moderate to good linear correlation with gold-standard MRI muscle mass measurements [R males: 0.68 (0.60–0.76); females: 0.65 (0.56–0.73)], after accounting for data imbalance. CMI discriminated muscle mass ≤2.5 standard deviations from the mean, with an area under the curve of 0.80 (95% CI:0.75–0.86) in males and 0.84 (95% CI:0.78–0.89) in females. CMI consistently decreased with increasing age, frailty and comorbidity. Over an 8-year follow-up, lower CMI was associated with higher morality: adjusted hazard ratio for the 25th vs 75th CMI centiles were 0.61 (95% CI: 0.58–0.66) in males and 0.72 (95% CI: 0.66–0.78) in females.

**Conclusions:**

In over 450 000 UK Biobank participants, low CMI was significantly associated with baseline comorbidity, frailty and survival on follow-up (independent of age, sex, and comorbidity). This study supports CMI as a potential biological signature for skeletal muscle wasting in clinical and research settings.

KEY LEARNING POINTS **What was known:**

- Skeletal muscle wasting is a well-known consequence of acute illness, associated with increased morbidity, mortality, and adverse long-term outcomes.
- Gold standard measurement of muscle is with magnetic resonance imaging (MRI).
- This is the first study investigating the relationship of Creatinine Muscle Index (CMI) with gold standard MRI muscle mass measurement.

**This study adds:**

- This study demonstrates a positive correlation between CMI and MRI muscle mass measurements.
- It strengthens the association of a low CMI with increasing age, frailty and comorbidity, as well as the association with higher mortality.

**Potential impact:**

- CMI is a potentially useful blood biomarker that can be used to quantify skeletal muscle mass in both clinical and research settings.

## INTRODUCTION

Skeletal muscle wasting and the associated decline in muscle function are hallmarks of aging, as well as acute and chronic disease [1], associated with increased morbidity, mortality, and substantial societal and economic cost [2–4]. Accelerated muscle wasting accompanies acute severe illness [5, 6] leading to impaired recovery and adverse long-term outcomes. Skeletal muscle wasting also affects those with chronic diseases, such as cardiovascular disease, cancer, and kidney disease, and is strongly associated with morbidity and adverse outcomes [7–10].

Despite clinical significance, muscle quantity and quality are rarely assessed in interventions targeting quality of life and independence. One explanation is that gold-standard methods, magnetic resonance imaging (MRI) or computed tomography, are costly and impractical [11, 12]. Consequently, both in clinical practice and research, skeletal muscle wasting is often overlooked, delaying early intervention. There is an unmet need for accessible biological signatures to screen for impaired muscle health in ambulant populations and after acute illness. Such tools could provide deeper insights into the interplay between skeletal muscle wasting, acute illness [6] and long-term conditions [13].

Creatinine muscle index (CMI), has been proposed as a biological signature for skeletal muscle wasting [14]. Derived from serum creatinine and cystatin C, CMI estimates creatinine generation from skeletal muscle [14, 15]. CMI only requires concurrent measurement of creatinine and cystatin C in a single blood sample, making it cost-effective and easy to implement in both research and clinical practice. Creatinine is released from skeletal muscle cells and is freely filtered by the glomerulus, therefore steady-state plasma concentration depends on both muscle mass and kidney function. Cystatin C, also principally excreted by glomerular filtration, is released from all nucleated cells providing an estimate of kidney function independent of muscle mass. The CMI equation estimates creatinine excretion rate normalized to body surface area, reflecting creatinine generation in steady state and serving as a surrogate of muscle mass. In a large longitudinal cohort, CMI has been associated with future mortality, morbidity, and frailty [14]. Similar findings have been shown using creatinine to cystatin C ratio, a cruder reflection of creatinine production relative to body size [16–19]. More recent work has shown a significant association of CMI with morbidity and mortality in the chronic kidney disease (CKD) population [20, 21].

To investigate the relationship between CMI and skeletal muscle mass, we analyzed data in over 450 000 UK Biobank participants. We assessed CMI against gold-standard imaging techniques, MRI and dual-energy X-ray absorptiometry (DEXA), then explored associations between CMI, comorbidities, frailty, and mortality—key outcomes linked with muscle wasting.

## MATERIALS AND METHODS

### Study population

UK Biobank recruited 502 180 participants between 2006 and 2010 collecting health questionnaires, physical measurements (including bioimpedance), and blood samples. The full recruitment process has been described [22]. Healthcare outcomes were obtained from self-reported data at recruitment and follow-up visits, linked electronic health records including primary care, hospital admissions, and national death registries. Ethical approval was granted by the North-West Multi-Centre Research Ethics Committee (REC Reference: 16/NW/0274) and all participants gave consent for use of their data (finalized: 24/11/2024). This study was conducted under UK Biobank application 102574.

Participants with baseline creatinine and cystatin C measurements were included. The MRI cohort included those with complete data on bilateral anterior, posterior, and total thigh fat-free muscle volume. The DEXA cohort included those with complete bilateral arm and leg fat-free mass data. The study flow sheet is in Supplemental Fig. S7.

### Definitions

Detailed variable definitions are in the supplement.

#### Creatinine muscle index

CMI estimates body surface area-normalized creatinine filtration, a surrogate of creatinine generation and muscle mass [14]. CMI was calculated using estimated GFR based on cystatin C (eGFRcys) [23], a muscle-independent measure of kidney function (Equation 1).


(1) \begin{eqnarray*}
CMI\left( {mg/day/1.73{m}^2} \right) &=& \mathit{eGFRcys}\left[ {ml/\min \,per\,1.73\,{m}^2} \right]\\&& \times \, \mathit{serum}\,\mathit{creatinine}\left[ {mg/dl} \right] \times 14.4
\end{eqnarray*}

#### Total thigh fat-free muscle volume (TTFMV)

Includes MRI-derived anterior and posterior thigh muscle volumes from both legs, normalized to body surface area (L/1.73 m^2^) for regression analysis.

#### DEXA appendicular lean mass (DEXA-ALM)

Calculated from combined appendicular lean mass and normalized to body surface area for regression analysis (kg/1.73 m^2^).

#### Demographics

Age was defined at first blood sampling and categorized into seven 5-year ranges: 38–43, 44–48, 49–53, 54–58, 59–63, 64–68, 69–73. Ethnicity was re-coded per Office of National Statistics grouping [24].

#### Sarcopenia

Sarcopenia was defined using the European Working Group on Sarcopenia in Older People (EWGSOP2) criteria [25]:

- Confirmed sarcopenia: Males (a): hand grip strength <27 kg and appendicular skeletal muscle/height^2^ <7 kg/m^2^, and Females (b): hand grip strength <16 kg and appendicular skeletal muscle/height^2^ <5.5 kg/m^2^.
- Probable sarcopenia: Males (c): hand grip strength <27 kg, and Females (d): hand grip strength <16 kg.

#### Comorbidities

Comorbidities (IHD, stroke, COPD, hypertension, diabetes, thyroid disease, and cancer) were identified using UK Biobank algorithmic outcomes, self-reported diagnosis, ICD-10, and ICD-9 codes (cross-checked with UK Biobank mappings). CKD stage G3-5 was identified using eGFRcr using the 2021 Chronic Kidney Disease Epidemiology Collaboration race-free equation [23].

Frailty and comorbidity were assessed using: UK Biobank Frailty Index [26], Hospital Frailty Risk Score (HFRS) [27], Charlson Comorbidity Index (CCI) [28], and the Fried Frailty Phenotype [29].

#### Survival

Follow-up was defined as 8 years from recruitment date or death, whichever occurred first.

### Data analysis

#### Missing data

All statistical analysis was carried out using a complete case analysis of each cohort. Within each cohort, the level of missingness was less than 5% per variable.

#### Statistical methods

Statistical analysis was carried out with R version 4.4.1 utilizing *rms, pROC, clinfun*, and *stats*. Categorical variables are presented as counts and percentages, continuous variables as median and interquartile range (IQR). Between group comparisons for continuous variables utilized the Wilcoxon-Mann-Whitney test and the χ^2^ test for categorical variables.

CMI was compared to BSA-normalized gold-standard measurements of muscle mass [total thigh fat-free muscle volume (TTFMV) & DEXA] using linear regression, with CMI as the predictor. Analyses were done with and without weighting, separately by sex and combined. 95% CI and regression lines were calculated using bootstrap resampling (1000 replicates).

As the UK Biobank recruited a predominantly healthy population, with a relatively narrow age range and low ethnic diversity, normalized muscle mass observations were tightly clustered particularly after normalization for size. This resulted in a large proportion of the variation between participants’ muscle mass or CMI being accounted for by measurement variation, rather than a biological difference in muscle. As measurement errors will not be correlated between different tests, clustering will tend to flatten the regression line and reduce the correlation coefficient even if there is a strong relationship between biomarkers and the biological variable in question. Several methods exist to deal with imbalanced datasets in regression problems. In our analysis we wished to assess the relationship between muscle mass and CMI across the biological range of muscle mass, i.e. to balance regression across the range of muscle mass in the cohort. To achieve this, we weighted patient values by the reciprocal of their local Kernel density estimate for the gold standard measure of muscle mass. This resulted in available data across the range of muscle mass having equal contributions to the regression. To avoid the over-influence of extreme outliers the top 10 most isolated values were assigned a maximal weighting. Weighted regression addressed the data imbalance within the dataset; the vast majority of datapoints represent closely clustered values of normal muscle mass whereas pathologically low muscle mass is relatively poorly represented. Model performance was assessed by root mean square error of residuals.

Sensitivity analyses included anterior and posterior FFMV, subgroups by age, BMI, and ethnicity. Receiver Operating Characteristic Area Under Curve (ROC-AUC) assessed the ability of CMI to discriminate measures of TTFMV 2 and 2.5 SD from the mean. Kaplan-Meir curves were used to examine survival over time by age, comorbidity, and CMI. Cox-Proportional Hazards models were constructed to determine the relationship between CMI and mortality, accounting for age and comorbidity using restricted cubic splines to model a non-linear relationship. Proportional hazards assumptions were checked using scaled Schoenfeld residual plots, non-proportionality was handled by stratification (age and CCI). Boxplots were used to display the relationship between CMI, comorbidity, and frailty. The Jonckheere-Terpstra test was used to compare across ordered categories.

## RESULTS

### Demographics

A total of 468 710 participants had baseline serum creatinine and cystatin C measurements. After missing variable exclusion, the final cohort consisted of 450 812 participants. At first visit, the median age was 57 years (IQR 50–63) for females and 58 years (IQR 50–64) for males. Ninety-five percent were of white ethnicity. Multi-morbidity was infrequent, a CCI of ≥3 was present in 1.3% of females and 1.1% of males. Baseline frailty risk (HFRS) was low; 2.4% of females and 2.7% of males were intermediate risk, <0.1% of females and 0.1% of males were high risk. The majority (54% females, 59% males) had a robust Fried frailty phenotype, pre-frailty was identified in 41% of females and 38% of males, 4.4% of females and 3.2% of males were frail. Table 1 presents whole cohort characteristics by sex.

**Table 1:** Whole cohort—participant characteristics (complete case analysis).

Characteristic	Female, *N* = 244 260	Male, *N* = 206 552
**Age, baseline visit (years), Median (IQR)**	57 (50, 63)	58 (50, 64)
**Ethnicity, *n* (%)**		
White	231 464 (95%)	195 351 (95%)
Black	3827 (1.6%)	2893 (1.4%)
Asian	3730 (1.5%)	4363 (2.1%)
Other	2979 (1.2%)	2139 (1.0%)
Mixed	1631 (0.7%)	991 (0.5%)
Unknown	629 (0.3%)	815 (0.4%)
**Smoking status, *n* (%)**		
Current	21 524 (8.8%)	25 332 (12%)
Previous	76 906 (31%)	79 342 (38%)
Never	145 026 (59%)	101 173 (49%)
Prefer not to answer	804 (0.3%)	705 (0.3%)
**Height (cm), Median (IQR)**	162 (158, 167)	176 (171, 180)
**Weight (kg), Median (IQR)**	69 (62, 79)	84 (76, 94)
**BMI (kg/m^2^), Median (IQR)**	26.1 (23.4, 29.6)	27.3 (25.0, 30.0)
**Body surface area (m^2^), Median (IQR)**	1.74 (1.65, 1.86)	2.01 (1.90, 2.12)
**Diet, *n* (%)**		
Omnivore	238 820 (98%)	203 744 (99%)
Vegetarian	5440 (2.2%)	2808 (1.4%)
**Handedness, *n* (%)**		
Left-handed	20 628 (8.4%)	21 407 (10%)
Right-handed	220 288 (90%)	180 793 (88%)
Use both right and left hands equally	3313 (1.4%)	4298 (2.1%)
Prefer not to answer	31 (<0.1%)	54 (<0.1%)
**Creatinine (mg/dL), Median (IQR)**	0.71 (0.64, 0.79)	0.90 (0.82, 1.00)
**Cystatin C (mg/L), Median (IQR)**	0.86 (0.78, 0.95)	0.92 (0.84, 1.01)
**eGFRcr, Median (IQR)**	97 (87, 104)	97 (87, 104)
**eGFRcys, Median (IQR)**	90 (78, 101)	88 (77, 100)
**eGFRcr-cys, Median (IQR)**	97 (86, 106)	96 (86, 105)
**Hospital frailty risk score, Median, (Min-Max)**	0.00 (0.00–43.80)	0.00 (0.00–38.60)
**Hospital frailty risk score level, *n* (%)**		
Low risk	238 174 (98%)	200 709 (97%)
Intermediate risk	5906 (2.4%)	5581 (2.7%)
High risk	180 (<0.1%)	262 (0.1%)
**Charlson comorbidity index, Median, (Min-Max)**	0.00 (0.00–14.00)	0.00 (0.00–15.00)
**Charlson comorbidity index level, *n* (%)**		
0	222 154 (91%)	189 941 (92%)
1–2	19 032 (7.8%)	14 296 (6.9%)
≥3	3074 (1.3%)	2315 (1.1%)
**UK Biobank frailty index, Median (IQR)**	0.11 (0.07, 0.17)	0.11 (0.07, 0.16)
**Frailty phenotype, *n* (%)**		
Robust	132 625 (54%)	121 283 (59%)
Pre-frail	100 930 (41%)	78 653 (38%)
Frail	10 705 (4.4%)	6616 (3.2%)
**Hypertension, *n* (%)**	57 881 (24%)	64 303 (31%)
**Ischaemic heart disease, *n* (%)**	7200 (2.9%)	15 958 (7.7%)
**Stroke, *n* (%)**	2679 (1.1%)	3685 (1.8%)
**Diabetes, *n* (%)**	8390 (3.4%)	14 042 (6.8%)
**CKD (G3–G5), *n* (%)**	3590 (1.5%)	3189 (1.5%)
**Cancer, *n* (%)**	26 598 (11%)	16 012 (7.8%)
**Thyroid disease, *n* (%)**	22 452 (9.2%)	4176 (2.0%)
**COPD, *n* (%)**	4139 (1.7%)	4429 (2.1%)

BMI, body mass index; eGFR, estimated glomerular filtration rate; eGFRcr, eGFR-creatinine; eGFRcys, eGFR-cystatin C; eGFRcr-cys, eGFR-creatinine cystatin C; CKD, chronic kidney disease; COPD, chronic obstructive pulmonary disease.

#### MRI and DEXA cohorts

The MRI case-control cohort includes 33 799 participants and the DEXA case-control cohort 34 728, with 25 727 represented in both. Participant characteristics mirrored the whole cohort (Supplemental Tables S3, S5). In the MRI cohort, median normalized TTFFMV was 8.11 L/1.73 m^2^ (IQR 7.80–8.66 L/1.73 m^2^) for females and 10.62 L/1.73 m^2^ (IQR 9.95–11.33 L/1.73 m^2^) for males. TTFMV and CMI declined with increasing age (Supplemental Table S6). In the DEXA cohort, median DEXA appendicular lean mass (DEXA-ALM) for females was 17.15 kg/1.73m^2^ (IQR 16.25–18.13 kg/1.73 m^2^) and 21.88 kg/1.73 m^2^ (20.71–23.08 kg/1.73 m^2^, *P* < .001) for males. Comorbidity and frailty levels were low at baseline.

### Association of CMI with MRI measured muscle volumes

Across all cohorts, CMI values were significantly greater in males than females (*P* < .001), as were all muscle mass measurements (*P* < .001) (Supplement Table S7). In the whole cohort, 3754 (0.83%) participants had a CMI ≤ 2SD below the mean, 304 (0.90%) in the MRI cohort and 291 (0.84%) in the DXA cohort.

**Figure 1:**
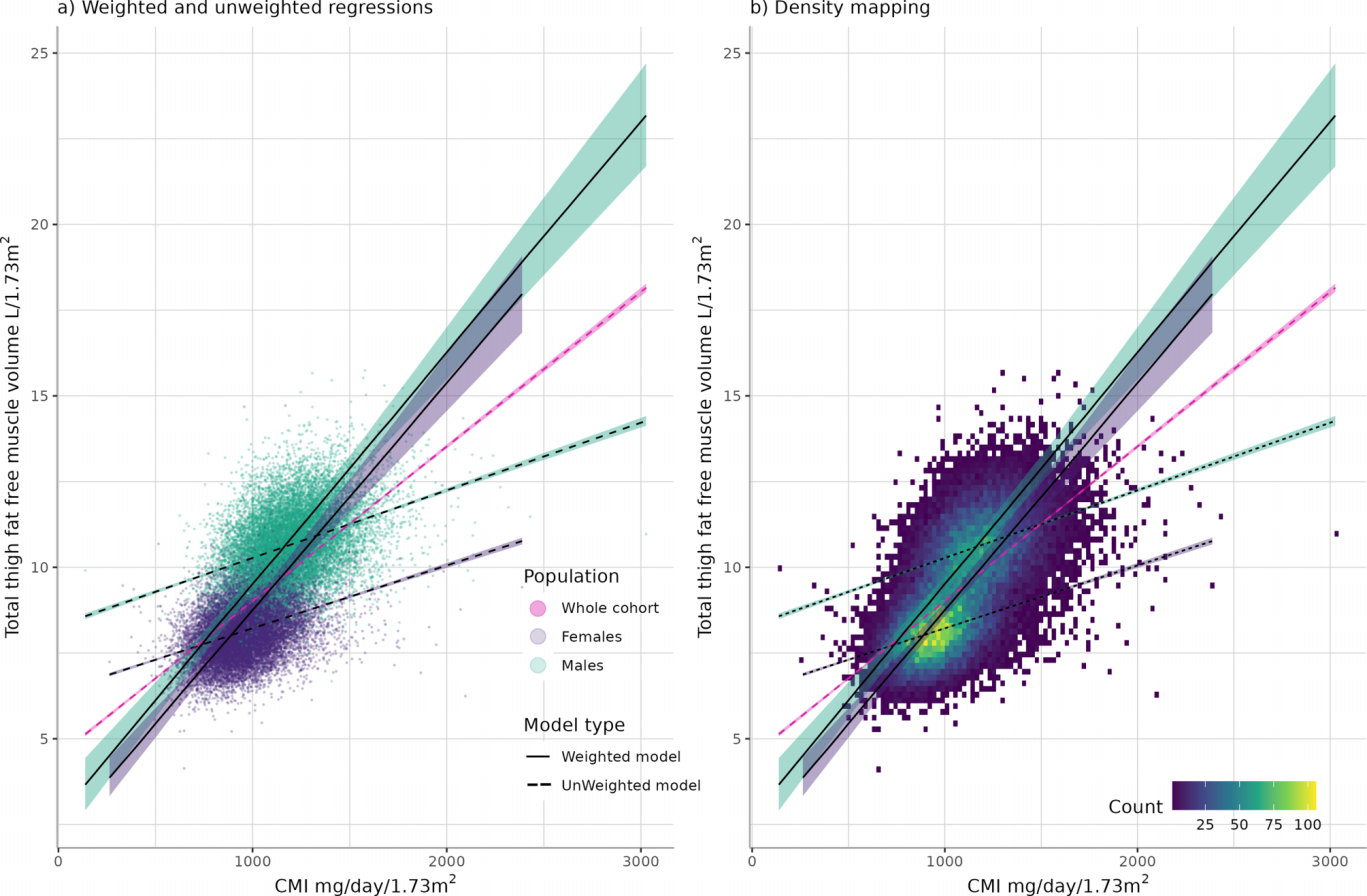
(a) Graph showing linear regression lines for both weighted and unweighted models within the whole cohort and for males and females separately. (b) Graph showing density mapping with regression lines overlying for weights and unweighted regression models and for the whole cohort, males and females separately.

Regression models illustrating the relationship between TTFMV (L/1.73 m^2^) and CMI (mg/day/1.73 m^2^) are shown in Fig. 1.

In the MRI cohort, unweighted linear regression modelling between TTFMV (L/1.73 m^2^) and CMI (mg/day/1.73 m^2^) showed moderate correlation [R 0.62 (0.61–0.62)]. Correlation worsened when stratified by sex, males R 0.37 (0.35–0.38) and females R 0.36 (0.35–0.38). To allow for the imbalanced dataset, weighted regression was used. R was 0.68 (0.60–0.76) and 0.65 (0.56–0.73) for males and females respectively, suggesting a moderate to good positive correlation between TTFMV and CMI (Table 2). Similar results were seen across anterior/posterior muscle compartments (Supplement Table S8) and sub-analyses by ethnicity, BMI categories, and age (Supplement Figs S1–S3). Weighted and unweighted regression modelling using DEXA-ALM in the DEXA cohort showed moderate positive correlation (Supplemental Table S9).

**Table 2:** Unweighted and weighted linear regression modelling within the MRI cohort.

	Outcome variable: total thigh fat free muscle volume (L)		
*Model*	*Coefficient (SE, p)*	*R (95% CI)*	*R^2^ (95% CI)*
Unweighted linear regression: Males and females combined	0.005 (3.13e−05, *P* < .001)	0.62 (0.61–0.62)	0.38 (0.37–0.39)
Unweighted linear regression: Males	0.002 (3.92e−05, *P* < .001)	0.37 (0.35–0.38)	0.14 (0.13–0.15)
Unweighted linear regression: Females	0.002 (3.53e−05, *P* < .001)	0.36 (0.35–0.38)	0.13 (0.12–0.14)
Weighted linear regression: Males	0.007 (5.74e−5, *P* < .001)	0.68 (0.60–0.76)	0.46 (0.34–0.58)
Weighted linear regression: Females	0.007 (5.88e−5, *P* < .001)	0.65 (0.56–0.73)	0.42 (0.28–0.54)

### The relationship of CMI with low muscle mass, comorbidity, frailty, and survival

#### Whole cohort: low muscle mass

The ROC-AUC for discrimination of TTFMV ≤ 2 SD from the mean was 0.74 (95% CI: 0.71–0.77) in males and 0.77 (95% CI: 0.74–0.80) in females. The AUC improved when discriminating TTFMV ≤ 2.5 SD from the mean, 0.80 (95% CI: 0.75–0.86) and 0.84 (95% CI: 0.78–0.89) in males and females respectively (Supplement Fig. S4). The ability of CMI to discriminate “confirmed” and “probable” sarcopenia, based on guideline definitions [25], showed moderate discriminative ability (Supplement Fig. S5).

#### Whole cohort: CMI and frailty markers

**Figure 2:**
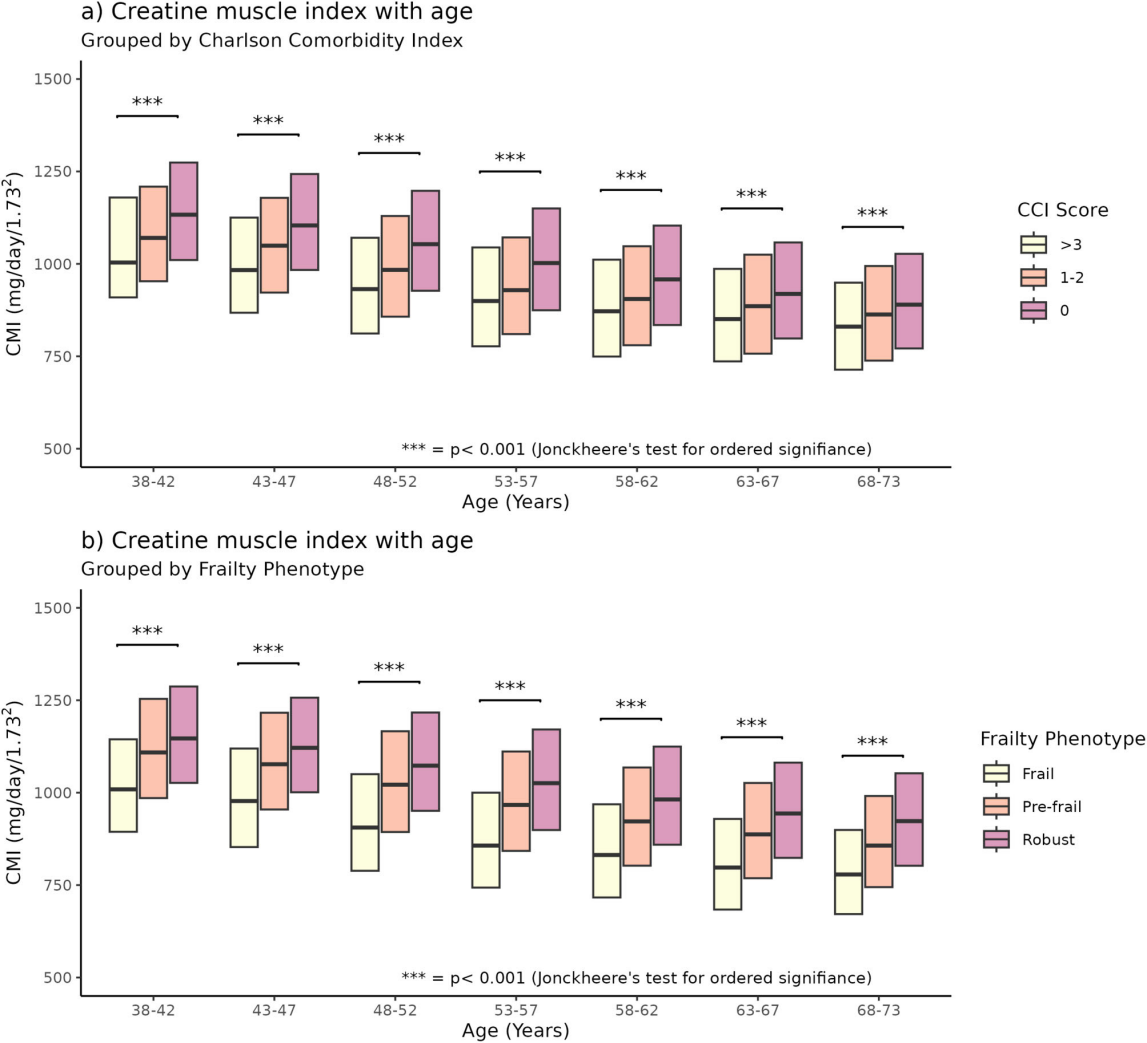
(a) Boxplots showing creatinine muscle index by age, grouped by Charlson Comorbidity Index. (b) Boxplots showing CMI by age, grouped by Fried frailty phenotype.

CMI decreased with increasing age, as well as with an increase in CCIand frailty (Fried frailty phenotype). Within each age group, the difference in CMI was significant (Fig. 2).

#### Whole cohort: CMI and mortality

**Figure 3:**
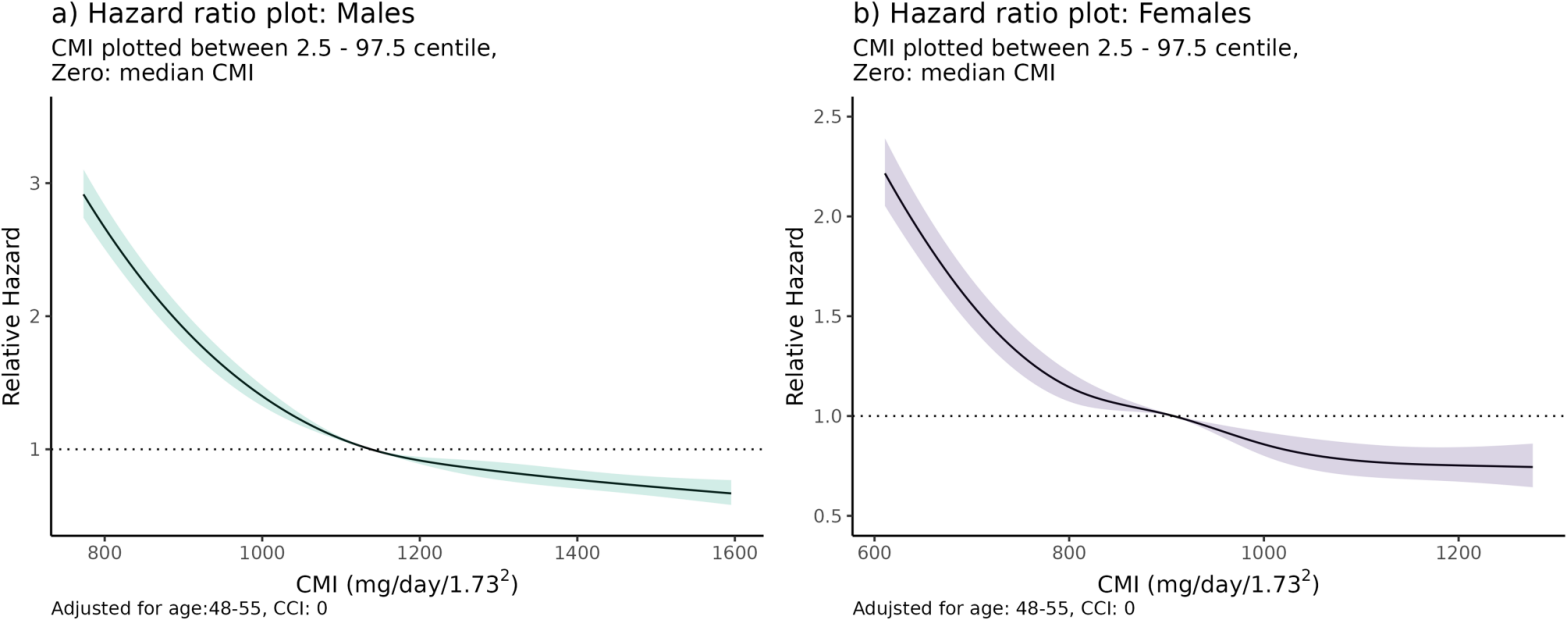
(a) Hazard ratio plot for creatinine muscle index (CMI) (males) adjusted for age (48–55) and Charlson Comorbidity Index (CCI) (0). (b) Hazard ratio plot for CMI (females) adjusted for age (48–55) and CCI (0).

During the 8-year follow-up period, 14 279 (3.3%) people died of any cause. When stratified for age and comorbidity, there was an association between increasing hazard of death with decreasing CMI for both sexes (Fig. 3). The association was non-linear with an inflection above the median CMI with a plateau of decreased risk at a higher CMI, but a steadily increasing risk for CMI below the median. With the very large sample size, the proportional hazards assumption was statistically violated, however inspection of residual plots (Supplement Fig. S6) demonstrated a consistent relationship between CMI and the hazard of death over time. Females demonstrated an age and comorbidity adjusted hazard ratio of 0.72 (95% CI: 0.66–0.78) for risk of death when comparing an individual at the 25th vs 75th centile of CMI. Males demonstrated a hazard ratio of 0.61 (95% CI: 0.58–0.66) for the risk of death, comparing individuals between at 25th vs 75th centiles of CMI.

## DISCUSSION

In this large UK population study, we found that CMI consistently correlated with gold-standard measurement of MRI muscle volume. This correlation was observed to a lesser degree using DEXA measured muscle mass. CMI had the expected relationship with age that you would find with muscle mass; this supports CMI as a potential muscle mass surrogate. Importantly, lower CMI was associated with increasing frailty, multi-morbidity and higher mortality, highlighting its potential utility as a marker of muscle wasting in acute illness and chronic disease, as well as functional outcomes in patients experiencing age- or illness-related muscle loss.

When males and females were considered separately using unweighted linear regression, the correlation between CMI and normalized MRI muscle volume was poor, with flattening of the regression line and a biologically unfeasible intercept. This reflects data clustering in a narrow range of “healthy” normalized muscle volumes, masking biological variation and limiting detection of any true CMI-skeletal muscle relationships. Weighted regression demonstrated better correlation across a clinically relevant range. Similar slopes were independently derived in both sexes, with biologically appropriate intercepts, where, as CMI tended to zero, so did MRI muscle volume. Models were similar in terms of gradient and intercept to a mixed-sex unweighted regression. CMI demonstrated moderate ability to discriminate low muscle mass. Poorer correlation with DEXA may be explained by underestimation of age-related muscle loss by up to 30% compared with MRI [30]. Across all age groups, CMI consistently declined with increasing comorbidity and frailty. Skeletal muscle wasting, along with loss of skeletal muscle function, are hallmarks of sarcopenia: a key component of the frailty phenotype. Muscle form defines function and this determines outcomes [31]. Lower CMI was independently associated with a higher risk of death, with a plateau in the protective effect of higher muscle mass above the median CMI. Whilst discrimination of sarcopenia using CMI was modest within a generally healthy population, its good correlation with long-term survival and frailty suggests it warrants further investigation in relevant patient populations, including those with CKD, as an index of functional muscle mass.

Our study is strengthened by the large sample size of over 450 000 participants for assessment of clinical outcomes and gold-standard measurements of muscle mass in over 30 000 participants. However, we acknowledge that in a large majority of patients there was a low level of chronic disease and ill-health. To accommodate this imbalance, we weighed our regression analysis as described. Importantly, CMI was able to discriminate participants with very low or high muscle mass, an analysis not dependent on weighting. A further limitation of this dataset is the time interval between blood sampling and MRI measurement (median = 9.1 years). A stronger correlation might be seen if these measures were made simultaneously. Given the described limitations of the UK Biobank cohort, further research in comorbid populations is required to confirm the utility of CMI as a screening tool for low muscle mass and sarcopenia in clinically relevant groups. This includes those with CKD, which is poorly resonated in the UK Biobank population, with only 1.5% of our total cohort having an estimated glomerular filtration rate (eGFR_Cr_) of ≤60 ml/min/1.73 m^2^. A multicentre study of people with CKD has shown a significant association of CMI with both markers of sarcopenia and mortality [20].

While CMI has been correlated with frailty and survival [14], we are not aware of any prior studies comparing CMI against gold-standard muscle mass measurements. Other indices, such as sarcopenia index [(SI, serum creatinine/serum cystatin C) × 100], have been assessed against CT imaging [18]. As a mathematic estimate of normalized creatinine generation, CMI represents the most interpretable and appropriate biochemical proxy of muscle mass. SI has been investigated within the UK Biobank [19] and was found to be a fair discriminator for sarcopenia based on BIA-ALM and handgrip strength (AUC 0.73 for males, 0.71 for females). However, this study did not utilize gold-standard measurements. Bio-impedance and handgrip strength are screening tools for low muscle mass and function with many potential biases. Another UK Biobank study [32] compared SI and MRI fat free muscle volume but showed inconsistent discriminative ability for identifying muscle volumes below the 20th percentile (AUC 0.57 for males, 0.62 for females). This study did not normalize MRI muscle volume to body size, neglecting the fact that SI, as an estimate of the ratio of creatinine to cystatin C production, like CMI, represents a relative not absolute measure of body muscle composition.

Several studies have demonstrated that relatively lower creatinine and higher cystatin C, assessed as a ratio or by discrepancies in eGFR, is associated with adverse outcomes and increased mortality, both in acute and chronic disease [33–36]. The ARIC cohort, a large-scale, long-term prospective study found that lower CMI was associated with frailty and increased mortality [14]. Our findings further demonstrate a correlation between CMI and gold-standard muscle measurement. We showed a robust association between CMI, frailty, comorbidity and risk of death. Together, these results add greater confidence to CMI as a screening tool for low muscle mass and its associated outcomes: the development of frailty and premature death. These results are also in line with recent meta-analysis which demonstrates that in the outpatient setting, the presence of eGFRcys at least 30% lower than eGFRcr was associated with significantly higher rates of all-cause mortality, cardiovascular events, and kidney failure, underlining the importance of lower CMI both as an indicator of sarcopenia and a flag of potential under-estimation of severity of CKD by serum creatinine [37].

The interpretation of CMI as a muscle mass measure assumes cystatin C accurately reflects kidney function. GFR-independent variations in cystatin C have been described in older age, male gender, greater weight, diabetes, smoking, and higher serum C-reactive protein [38]. One suggested mechanism is the presence of chronic inflammation [39], whilst others hypothesize a reduction in fractional glomerular clearance in early glomerular disease [40, 41]. Kleeman et al. have associated increased cystatin C relative to creatinine with inflammation, glucocorticoid signalling, and immune dysregulation [42]. However, estimation of cystatin C production was inferential and variation in muscle mass and creatinine production was not well-accounted for [42]. Without direct measurement of cystatin C production, or gold-standard GFR measurement, the exact contribution of elevated cystatin C production or reduced clearance is difficult to gauge. In studies with independent measurement of GFR, both lower creatinine and higher cystatin C demonstrated association with outcomes independent of true kidney function [43]. It is possible that elevated CMI represents a combination of low muscle mass and an adverse immuno-inflammatory state. If so, this consolidates CMIs’ strength as a biological signature, reflecting the biology of muscle mass maintenance, the process of muscle wasting and an underpinning mechanism leading to development of frailty and increased risk of death. Further research encompassing this is therefore required to examine the utility of CMI in higher-risk populations; accurate measurement of GFR in patients with abnormal CMI would allow quantification of independent contributions from both muscle mass and pro-inflammatory states.

In conclusion, CMI could be an effective, simple and useful biological signature for screening individuals at risk of low muscle mass. CMI has shown a consistent correlation with adverse outcomes of muscle loss including frailty and risk of death. CMI may also serve as a surrogate endpoint for early phase interventional studies assessing muscle-targeted interventions in chronic disease or after acute illness and as a screening tool for identifying potential muscle loss in the community and after recovery from acute illness.

## Supplementary Material

sfag139_Supplemental_File

## Data Availability

This study has been conducted under the UK Biobank application code 102574. UK Biobank data is available for healthcare researchers on application at https://www.ukbiobank.ac.uk/.
