# Supplementary material for: Creatinine muscle index in UK Biobank: comparison with MRI and associations with frailty and mortality
Source: Clin Kidney J. 2026 May 9;19(5):sfag139. doi: 10.1093/ckj/sfag139 (PMC13184687; doi:10.1093/ckj/sfag139)
Supplement: sfag139_Supplemental_File [file sfag139_supplemental_file.docx]

**Supplementary information for Creatinine Muscle Index in UK Biobank: Comparison with MRI and associations with Frailty and Mortality**

Giada Azzopardi^1,2^, Thomas Davies^1,2^, Myles J. Lewis^3^, Zudin Puthucheary^1,2^ & John R. Prowle^1,2^

1. Adult Critical Care Unit, The Royal London Hospital, Barts Health NHS Trust, Whitechapel Road, London, E1 1FR, UK

2. Critical Care and Peri-operative Medicine Research Group, William Harvey Research Institute, Barts and the London School of Medicine and Dentistry, Queen Mary University of London, London, UK

3. Centre for Centre for Experimental Medicine and Rheumatology, William Harvey Research Institute, Barts and the London School of Medicine and Dentistry, Queen Mary University of London, London, UK

**Correspondence:**

Dr. Giada Azzopardi

Adult Critical Care Unit,

The Royal London Hospital,

Barts Health NHS Trust,

Whitechapel Road,

London E1 1BB,

United Kingdom

Tel: 020 3594 0410

Email: giada.azzopardi@nhs.net

**Contents**

1. **Participant characteristic tables**

Table S1: Whole cohort

Table S2: Magnetic resonance imaging (MRI) cohort

Table S3: MRI cohort (case control)

Table S4: Dual energy X-ray absorptiometry (DEXA) cohort

Table S5: DEXA cohort (case control)

1. **Total thigh fat-free muscle volume (TTFMV) and creatinine muscle index (CMI) by age category**

Table S6: Median TTFMV and CMI by age category

1. **Outcome variables by cohort**

Table S7: Outcome variables by cohort

1. **Regression models using anterior and posterior muscle compartment volumes**

Table S8: Regression models using anterior and posterior muscle compartment volumes

1. **Sensitivity analysis: Ethnicity, Age and body mass index (BMI)**

Figure S1: Regression lines split by ethnicity

Figure S2: Regression lines split by age

Figure S3: Regression lines split by BMI category

1. **Regression modelling in the DEXA cohort**

Table S9: Unweighted and weighted regression modelling in the DXA cohort [outcome variables: DEXA-appendicular lean mass (DEXA-ALM)

1. **Receiver Operating Characteristic (ROC) curves for TTFFMV ≤2 SD and ≤2.5 SD below the mean using CMI**

Figure S4: ROC curve for detection of TTFMV ≤2 and ≤2.5 SD below the mean using CMI

1. **ROC curves for sarcopenia detection as per European Working Group on Sarcopenia in Older People (EWGSOP) guideline definitions using CMI**

Figure S5: Graph showing ROC curves for the detection of confirmed and probable sarcopenia using CMI

1. **Shoenfield residuals plots**

Figure S6: Scaled Shoenfield residuals plots for Cox-proportional hazards models

1. **Cohort selection process**

Figure S7: Cohort selection process

1. **Detailed variable definitions**
2. **Comorbidity coding**
3. **Approach to linear regression analysis**
4. **References**

**1. Participant characteristics tables**

Table S1: Whole cohort – Participant characteristics

| **Characteristic** | **Overall**  N = 468,710 | **Female**  N = 254,138 | **Male**  N = 214,572 |
| --- | --- | --- | --- |
| **Age, Baseline visit (years), Median (IQR)** | 58 (50, 63) | 57 (50, 63) | 58 (50, 64) |
| **Ethnicity, n (%)** |  |  |  |
| White | 441,771 (94%) | 239,811 (94%) | 201,960 (94%) |
| Black | 7,285 (1.6%) | 4,158 (1.6%) | 3,127 (1.5%) |
| Asian | 9,089 (1.9%) | 4,170 (1.6%) | 4,919 (2.3%) |
| Other | 5,616 (1.2%) | 3,274 (1.3%) | 2,342 (1.1%) |
| Mixed | 2,746 (0.6%) | 1,710 (0.7%) | 1,036 (0.5%) |
| Unknown | 1,714 (0.4%) | 745 (0.3%) | 969 (0.5%) |
| Missing | 489 (0.1%) | 270 (0.1%) | 219 (0.1%) |
| **Smoking status, n (%)** |  |  |  |
| Current | 49,197 (11%) | 22,558 (8.9%) | 26,639 (12%) |
| Previous | 161,902 (35%) | 79,642 (31%) | 82,260 (38%) |
| Never | 255,234 (55%) | 150,680 (59%) | 104,554 (49%) |
| Prefer not to answer | 1,890 (0.4%) | 989 (0.4%) | 901 (0.4%) |
| Missing | 487 | 269 | 218 |
| **Height (cm), Median (IQR)** | 168 (162, 175) | 162 (158, 167) | 176 (171, 180) |
| Missing | 1,363 | 614 | 749 |
| **Weight (kg), Median (IQR)** | 76 (67, 88) | 69 (62, 79) | 84 (76, 94) |
| Missing | 1,563 | 797 | 766 |
| **BMI (kg/m^2^), Median (IQR)** | 26.7 (24.1, 29.9) | 26.1 (23.4, 29.7) | 27.3 (25.0, 30.1) |
| Missing | 1,871 | 886 | 985 |
| **Body surface area (m^2^), Median (IQR)** | 1.87 (1.72, 2.02) | 1.74 (1.65, 1.86) | 2.01 (1.90, 2.12) |
| Missing | 1,871 | 886 | 985 |
| **Diet, n (%)** |  |  |  |
| Omnivore | 459,555 (98%) | 248,150 (98%) | 211,405 (99%) |
| Vegetarian | 8,666 (1.9%) | 5,718 (2.3%) | 2,948 (1.4%) |
| Missing | 489 | 270 | 219 |
| **Handedness, n (%)** |  |  |  |
| Left-handed | 43,679 (9.3%) | 21,428 (8.4%) | 22,251 (10%) |
| Right-handed | 416,240 (89%) | 228,821 (90%) | 187,419 (87%) |
| Use both right and left hands equally | 8,054 (1.7%) | 3,516 (1.4%) | 4,538 (2.1%) |
| Prefer not to answer | 248 (<0.1%) | 103 (<0.1%) | 145 (<0.1%) |
| Missing | 489 | 270 | 219 |
| **Creatinine (mg/dL), Median (IQR)** | 0.80 (0.69, 0.92) | 0.71 (0.64, 0.79) | 0.90 (0.82, 1.00) |
| **Cystatin C (mg/L), Median (IQR)** | 0.89 (0.80, 0.98) | 0.86 (0.78, 0.95) | 0.92 (0.84, 1.01) |
| **eGFRcr, Median (IQR)** | 97 (87, 104) | 97 (87, 104) | 97 (87, 104) |
| **eGFRcys, Median (IQR)** | 89 (77, 101) | 90 (78, 101) | 88 (77, 100) |
| **eGFRcr-cys, Median (IQR)** | 96 (86, 106) | 97 (86, 106) | 95 (86, 105) |
| **Hospital Frailty Risk Score, Median, (Min - Max)** | 0.00 (0.00 - 43.80) | 0.00 (0.00 - 43.80) | 0.00 (0.00 - 38.60) |
| **Hospital Frailty Risk Score Level, n (%)** |  |  |  |
| High Risk | 631 (0.1%) | 263 (0.1%) | 368 (0.2%) |
| Intermediate Risk | 13,063 (2.8%) | 6,665 (2.6%) | 6,398 (3.0%) |
| Low Risk | 455,016 (97%) | 247,210 (97%) | 207,806 (97%) |
| **Charlson Comorbidity Index, Median, (Min - Max)** | 0 (0 - 15) | 0 (0 - 14) | 0 (0 - 15) |
| **Charlson Comorbidity Index Level, n (%)** |  |  |  |
| 0 | 426,693 (91%) | 230,357 (91%) | 196,336 (92%) |
| 1-2 | 36,044 (7.7%) | 20,440 (8.0%) | 15,604 (7.3%) |
| >3 | 5,973 (1.3%) | 3,341 (1.3%) | 2,632 (1.2%) |
| **Frailty Index, Median (IQR)** | 0.11 (0.07, 0.17) | 0.12 (0.07, 0.17) | 0.11 (0.07, 0.16) |
| Missing | 1,607 | 839 | 768 |
| **Frailty Phenotype, n (%)** |  |  |  |
| Robust | 257,518 (56%) | 134,471 (54%) | 123,047 (58%) |
| Pre-frail | 183,535 (40%) | 103,046 (41%) | 80,489 (38%) |
| Frail | 18,205 (4.0%) | 11,173 (4.5%) | 7,032 (3.3%) |
| Missing | 9,452 | 5,448 | 4,004 |
| **Hypertension, n (%)** | 128,786 (27%) | 61,130 (24%) | 67,656 (32%) |
| **Ischaemic heart disease, n (%)** | 25,277 (5.4%) | 7,936 (3.1%) | 17,341 (8.1%) |
| **Stroke, n (%)** | 7,251 (1.5%) | 3,043 (1.2%) | 4,208 (2.0%) |
| **Diabetes, n (%)** | 24,312 (5.2%) | 9,137 (3.6%) | 15,175 (7.1%) |
| **CKD (G3-G5), n (%)** | 7,356 (1.6%) | 3,891 (1.5%) | 3,465 (1.6%) |
| **Cancer, n (%)** | 44,529 (9.5%) | 27,838 (11%) | 16,691 (7.8%) |
| **Thyroid disease, n (%)** | 28,013 (6.0%) | 23,577 (9.3%) | 4,436 (2.1%) |
| **COPD, n (%)** | 9,310 (2.0%) | 4,490 (1.8%) | 4,820 (2.2%) |
| *Body mass index (BMI), estimated glomerular filtration rate (eGFR), eGFR-creatinine (eGFRcr), eGFR-cystatin C (eGFRcys), eGFR-creatinine cystatin C (eGFRcr-cys), chronic kidney disease (CKD), chronic obstructive pulmonary disease (COPD* | | | |

Table S2: MRI cohort – Participant characteristics

| **Characteristic** | **Overall**  N = 36,823 | **Female**  N = 19,293 | **Male**  N = 17,530 |
| --- | --- | --- | --- |
| **Age, Baseline visit (years), Median (IQR)** | 56 (49, 61) | 55 (48, 60) | 57 (50, 62) |
| **Ethnicity, n (%)** |  |  |  |
| White | 35,588 (97%) | 18,672 (97%) | 16,916 (96%) |
| Black | 258 (0.7%) | 143 (0.7%) | 115 (0.7%) |
| Asian | 428 (1.2%) | 160 (0.8%) | 268 (1.5%) |
| Other | 294 (0.8%) | 173 (0.9%) | 121 (0.7%) |
| Mixed | 154 (0.4%) | 102 (0.5%) | 52 (0.3%) |
| Unknown | 92 (0.2%) | 37 (0.2%) | 55 (0.3%) |
| Missing | 9 (<0.1%) | 6 (<0.1%) | 3 (<0.1%) |
| **Smoking status, n (%)** |  |  |  |
| Current | 2,294 (6.2%) | 993 (5.1%) | 1,301 (7.4%) |
| Previous | 12,101 (33%) | 5,848 (30%) | 6,253 (36%) |
| Never | 22,346 (61%) | 12,409 (64%) | 9,937 (57%) |
| Prefer not to answer | 73 (0.2%) | 37 (0.2%) | 36 (0.2%) |
| Missing | 9 (<0.1%) | 6 (<0.1%) | 3 (<0.1%) |
| **Height (cm), Median (IQR)** | 169 (163, 176) | 163 (159, 167) | 176 (172, 181) |
| Missing | 30 | 15 | 15 |
| **Weight (kg), Median (IQR)** | 75 (66, 86) | 67 (61, 76) | 83 (76, 92) |
| Missing | 39 | 21 | 18 |
| **BMI (kg/m^2^), Median (IQR)** | 26.0 (23.6, 28.8) | 25.2 (22.9, 28.4) | 26.7 (24.6, 29.1) |
| Missing | 47 | 25 | 22 |
| **Body surface area (m^2^), Median (IQR)** | 1.86 (1.72, 2.01) | 1.73 (1.64, 1.84) | 2.00 (1.90, 2.11) |
| Missing | 47 | 25 | 22 |
| **Diet, n (%)** |  |  |  |
| Omnivore | 35,979 (98%) | 18,743 (97%) | 17,236 (98%) |
| Vegetarian | 835 (2.3%) | 544 (2.8%) | 291 (1.7%) |
| Missing | 9 (<0.1%) | 6 (<0.1%) | 3 (<0.1%) |
| **Handedness, n (%)** |  |  |  |
| Left-handed | 3,439 (9.3%) | 1,607 (8.3%) | 1,832 (10%) |
| Right-handed | 32,801 (89%) | 17,442 (90%) | 15,359 (88%) |
| Use both right and left hands equally | 566 (1.5%) | 236 (1.2%) | 330 (1.9%) |
| Prefer not to answer | 8 (<0.1%) | 2 (<0.1%) | 6 (<0.1%) |
| Missing | 9 (<0.1%) | 6 (<0.1%) | 3 (<0.1%) |
| **Creatinine (mg/dL), Median (IQR)** | 0.80 (0.70, 0.92) | 0.71 (0.65, 0.79) | 0.91 (0.83, 1.00) |
| **Cystatin C (mg/L), Median (IQR)** | 0.86 (0.79, 0.94) | 0.83 (0.76, 0.91) | 0.90 (0.83, 0.97) |
| **eGFRcr, Median (IQR)** | 98 (89, 104) | 99 (89, 105) | 98 (88, 104) |
| **eGFRcys, Median (IQR)** | 93 (82, 103) | 96 (84, 104) | 91 (81, 102) |
| **eGFRcr-cys, Median (IQR)** | 99 (90, 107) | 100 (90, 108) | 98 (89, 106) |
| **Hospital Frailty Risk Score, Median, (Min - Max)** | 0.00 (0.00 - 16.90) | 0.00 (0.00 - 16.90) | 0.00 (0.00 - 16.20) |
| **Hospital Frailty Risk Score Level, n (%)** |  |  |  |
| High Risk | 5 (<0.1%) | 4 (<0.1%) | 1 (<0.1%) |
| Intermediate Risk | 437 (1.2%) | 241 (1.2%) | 196 (1.1%) |
| Low Risk | 36,381 (99%) | 19,048 (99%) | 17,333 (99%) |
| **Charlson Comorbidity Index, Median, (Min - Max)** | 0 (0 - 9) | 0 (0 - 9) | 0 (0 - 9) |
| **Charlson Comorbidity Index Level, n (%)** |  |  |  |
| 0 | 34,936 (95%) | 18,231 (94%) | 16,705 (95%) |
| 1-2 | 1,708 (4.6%) | 946 (4.9%) | 762 (4.3%) |
| >3 | 179 (0.5%) | 116 (0.6%) | 63 (0.4%) |
| **Frailty Index, Median (IQR)** | 0.09 (0.06, 0.14) | 0.10 (0.06, 0.14) | 0.09 (0.06, 0.14) |
| Missing | 23 | 13 | 10 |
| **Frailty Phenotype, n (%)** |  |  |  |
| Robust | 23,939 (65%) | 12,155 (63%) | 11,784 (67%) |
| Pre-frail | 12,146 (33%) | 6,656 (34%) | 5,490 (31%) |
| Frail | 483 (1.3%) | 330 (1.7%) | 153 (0.9%) |
| Missing | 255 (0.7%) | 152 (0.8%) | 103 (0.6%) |
| **Hypertension, n (%)** | 7,165 (19%) | 3,018 (16%) | 4,147 (24%) |
| **Ischaemic heart disease, n (%)** | 1,048 (2.8%) | 272 (1.4%) | 776 (4.4%) |
| **Stroke, n (%)** | 258 (0.7%) | 108 (0.6%) | 150 (0.9%) |
| **Diabetes, n (%)** | 940 (2.6%) | 319 (1.7%) | 621 (3.5%) |
| **CKD (G3-G5), n (%)** | 232 (0.6%) | 130 (0.7%) | 102 (0.6%) |
| **Cancer, n (%)** | 2,624 (7.1%) | 1,599 (8.3%) | 1,025 (5.8%) |
| **Thyroid disease, n (%)** | 1,743 (4.7%) | 1,463 (7.6%) | 280 (1.6%) |
| **COPD, n (%)** | 282 (0.8%) | 108 (0.6%) | 174 (1.0%) |
| *Body mass index (BMI), estimated glomerular filtration rate (eGFR), eGFR-creatinine (eGFRcr), eGFR-cystatin C (eGFRcys), eGFR-creatinine cystatin C (eGFRcr-cys), chronic kidney disease (CKD), chronic obstructive pulmonary disease (COPD* | | | |

Table S3: MRI cohort – Participant characteristics (case control)

| **Characteristic** | **Overall**  N = 33,799 | **Female**  N = 17,611 | **Male**  N = 16,188 |
| --- | --- | --- | --- |
| **Age, Baseline visit (years), Median (IQR)** | 55 (49, 61) | 55 (48, 60) | 57 (50, 62) |
| **Ethnicity, n (%)** |  |  |  |
| White | 32,693 (97%) | 17,066 (97%) | 15,627 (97%) |
| Black | 221 (0.7%) | 118 (0.7%) | 103 (0.6%) |
| Asian | 386 (1.1%) | 139 (0.8%) | 247 (1.5%) |
| Other | 266 (0.8%) | 154 (0.9%) | 112 (0.7%) |
| Mixed | 148 (0.4%) | 98 (0.6%) | 50 (0.3%) |
| Unknown | 85 (0.3%) | 36 (0.2%) | 49 (0.3%) |
| **Smoking status, n (%)** |  |  |  |
| Current | 2,103 (6.2%) | 900 (5.1%) | 1,203 (7.4%) |
| Previous | 11,063 (33%) | 5,304 (30%) | 5,759 (36%) |
| Never | 20,570 (61%) | 11,375 (65%) | 9,195 (57%) |
| Prefer not to answer | 63 (0.2%) | 32 (0.2%) | 31 (0.2%) |
| **Height (cm), Median (IQR)** | 169 (163, 176) | 163 (159, 167) | 176 (172, 181) |
| **Weight (kg), Median (IQR)** | 75 (66, 86) | 67 (61, 76) | 83 (76, 92) |
| **BMI (kg/m^2^), Median (IQR)** | 26.0 (23.7, 28.8) | 25.2 (22.9, 28.4) | 26.7 (24.6, 29.1) |
| **Body surface area (m^2^), Median (IQR)** | 1.87 (1.72, 2.01) | 1.73 (1.64, 1.84) | 2.00 (1.90, 2.11) |
| **Diet, n (%)** |  |  |  |
| Omnivore | 33,031 (98%) | 17,115 (97%) | 15,916 (98%) |
| Vegetarian | 768 (2.3%) | 496 (2.8%) | 272 (1.7%) |
| **Handedness, n (%)** |  |  |  |
| Left-handed | 3,151 (9.3%) | 1,465 (8.3%) | 1,686 (10%) |
| Right-handed | 30,133 (89%) | 15,926 (90%) | 14,207 (88%) |
| Use both right and left hands equally | 510 (1.5%) | 218 (1.2%) | 292 (1.8%) |
| Prefer not to answer | 5 (<0.1%) | 2 (<0.1%) | 3 (<0.1%) |
| **Creatinine (mg/dL), Median (IQR)** | 0.80 (0.70, 0.92) | 0.71 (0.65, 0.79) | 0.91 (0.83, 1.00) |
| **Cystatin C (mg/L), Median (IQR)** | 0.86 (0.79, 0.94) | 0.83 (0.76, 0.91) | 0.90 (0.83, 0.97) |
| **eGFRcr, Median (IQR)** | 98 (89, 104) | 99 (89, 105) | 98 (88, 104) |
| **eGFRcys, Median (IQR)** | 94 (83, 103) | 96 (84, 104) | 91 (81, 103) |
| **eGFRcr-cys, Median (IQR)** | 99 (90, 107) | 100 (90, 108) | 98 (89, 106) |
| **Hospital Frailty Risk Score, Median, (Min - Max)** | 0.00 (0.00 - 16.60) | 0.00 (0.00 - 16.60) | 0.00 (0.00 - 16.20) |
| **Hospital Frailty Risk Score Level, n (%)** |  |  |  |
| High Risk | 3 (<0.1%) | 2 (<0.1%) | 1 (<0.1%) |
| Intermediate Risk | 385 (1.1%) | 211 (1.2%) | 174 (1.1%) |
| Low Risk | 33,411 (99%) | 17,398 (99%) | 16,013 (99%) |
| **Charlson Comorbidity Index, Median, (Min - Max)** | 0 (0 - 9) | 0 (0 - 9) | 0 (0 - 9) |
| **Charlson Comorbidity Index Level, n (%)** |  |  |  |
| 0 | 32,080 (95%) | 16,644 (95%) | 15,436 (95%) |
| 1-2 | 1,557 (4.6%) | 860 (4.9%) | 697 (4.3%) |
| >3 | 162 (0.5%) | 107 (0.6%) | 55 (0.3%) |
| **Frailty Index, Median (IQR)** | 0.09 (0.06, 0.14) | 0.10 (0.06, 0.14) | 0.09 (0.05, 0.14) |
| **Frailty Phenotype, n (%)** |  |  |  |
| Robust | 22,227 (66%) | 11,238 (64%) | 10,989 (68%) |
| Pre-frail | 11,158 (33%) | 6,086 (35%) | 5,072 (31%) |
| Frail | 414 (1.2%) | 287 (1.6%) | 127 (0.8%) |
| **Hypertension, n (%)** | 6,503 (19%) | 2,738 (16%) | 3,765 (23%) |
| **Ischaemic heart disease, n (%)** | 932 (2.8%) | 242 (1.4%) | 690 (4.3%) |
| **Stroke, n (%)** | 231 (0.7%) | 94 (0.5%) | 137 (0.8%) |
| **Diabetes, n (%)** | 841 (2.5%) | 273 (1.6%) | 568 (3.5%) |
| **CKD (G3-G5), n (%)** | 199 (0.6%) | 110 (0.6%) | 89 (0.5%) |
| **Cancer, n (%)** | 2,400 (7.1%) | 1,456 (8.3%) | 944 (5.8%) |
| **Thyroid disease, n (%)** | 1,585 (4.7%) | 1,325 (7.5%) | 260 (1.6%) |
| **COPD, n (%)** | 241 (0.7%) | 89 (0.5%) | 152 (0.9%) |
| *Body mass index (BMI), estimated glomerular filtration rate (eGFR), eGFR-creatinine (eGFRcr), eGFR-cystatin C (eGFRcys), eGFR-creatinine cystatin C (eGFRcr-cys), chronic kidney disease (CKD), chronic obstructive pulmonary disease (COPD* | | | |

Table S4: DEXA cohort – Participant characteristics

| **Characteristic** | **Overall**  N = 36,689 | **Female**  N = 18,882 | **Male**  N = 17,807 |
| --- | --- | --- | --- |
| **Age, Baseline visit (years), Median (IQR)** | 56 (49, 61) | 55 (48, 61) | 57 (50, 62) |
| **Ethnicity, n (%)** |  |  |  |
| White | 35,294 (96%) | 18,163 (96%) | 17,131 (96%) |
| Black | 286 (0.8%) | 158 (0.8%) | 128 (0.7%) |
| Asian | 474 (1.3%) | 183 (1.0%) | 291 (1.6%) |
| Mixed | 193 (0.5%) | 130 (0.7%) | 63 (0.4%) |
| Other | 332 (0.9%) | 196 (1.0%) | 136 (0.8%) |
| Unknown | 102 (0.3%) | 47 (0.2%) | 55 (0.3%) |
| Missing | 8 (<0.1%) | 5 (<0.1%) | 3 (<0.1%) |
| **Smoking status, n (%)** |  |  |  |
| Current | 2,359 (6.4%) | 1,025 (5.4%) | 1,334 (7.5%) |
| Previous | 12,278 (33%) | 5,845 (31%) | 6,433 (36%) |
| Never | 21,964 (60%) | 11,967 (63%) | 9,997 (56%) |
| Prefer not to answer | 80 (0.2%) | 40 (0.2%) | 40 (0.2%) |
| Missing | 8 | 5 | 3 |
| **Height (cm), Median (IQR)** | 169 (163, 177) | 163 (159, 168) | 177 (172, 181) |
| Missing | 32 | 16 | 16 |
| **Weight (kg), Median (IQR)** | 76 (66, 86) | 68 (61, 76) | 83 (76, 92) |
| Missing | 42 | 25 | 17 |
| **BMI (kg/m^2^), Median (IQR)** | 26.1 (23.7, 28.9) | 25.2 (22.9, 28.5) | 26.8 (24.6, 29.2) |
| Missing | 49 | 28 | 21 |
| **Body surface area (m^2^), Median (IQR)** | 1.87 (1.72, 2.02) | 1.73 (1.64, 1.84) | 2.00 (1.90, 2.11) |
| Missing | 49 | 28 | 21 |
| **Diet, n (%)** |  |  |  |
| Omnivore | 35,827 (98%) | 18,314 (97%) | 17,513 (98%) |
| Vegetarian | 854 (2.3%) | 563 (3.0%) | 291 (1.6%) |
| Missing | 8 | 5 | 3 |
| **Handedness, n (%)** |  |  |  |
| Left-handed | 3,498 (9.5%) | 1,651 (8.7%) | 1,847 (10%) |
| Right-handed | 32,601 (89%) | 16,986 (90%) | 15,615 (88%) |
| Use both right and left hands equally | 576 (1.6%) | 239 (1.3%) | 337 (1.9%) |
| Prefer not to answer | 6 (<0.1%) | 1 (<0.1%) | 5 (<0.1%) |
| Missing | 8 | 5 | 3 |
| **Creatinine (mg/dL), Median (IQR)** | 0.81 (0.70, 0.92) | 0.71 (0.65, 0.79) | 0.91 (0.83, 1.00) |
| **Cystatin C (mg/L), Median (IQR)** | 0.86 (0.79, 0.95) | 0.83 (0.76, 0.91) | 0.90 (0.83, 0.98) |
| **eGFRcr, Median (IQR)** | 98 (88, 104) | 99 (88, 105) | 97 (88, 104) |
| **eGFRcys, Median (IQR)** | 93 (82, 103) | 95 (83, 104) | 91 (81, 102) |
| **eGFRcr-cys, Median (IQR)** | 99 (89, 107) | 100 (90, 108) | 97 (88, 106) |
| **Hospital Frailty Risk Score, Median, (Min - Max)** | 0.00 (0.00 - 16.80) | 0.00 (0.00 - 16.80) | 0.00 (0.00 - 16.10) |
| **Hospital Frailty Risk Score Level, n (%)** |  |  |  |
| High Risk | 3 (<0.1%) | 2 (<0.1%) | 1 (<0.1%) |
| Intermediate Risk | 426 (1.2%) | 227 (1.2%) | 199 (1.1%) |
| Low Risk | 36,260 (99%) | 18,653 (99%) | 17,607 (99%) |
| **Charlson Comorbidity Index, Median, (Min - Max)** | 0 (0 - 9) | 0 (0 - 9) | 0 (0 - 9) |
| **Charlson Comorbidity Index Level, n (%)** |  |  |  |
| 0 | 34,826 (95%) | 17,853 (95%) | 16,973 (95%) |
| 1-2 | 1,677 (4.6%) | 912 (4.8%) | 765 (4.3%) |
| >3 | 186 (0.5%) | 117 (0.6%) | 69 (0.4%) |
| **Frailty Index, Median (IQR)** | 0.10 (0.06, 0.14) | 0.10 (0.06, 0.14) | 0.09 (0.06, 0.14) |
| Missing | 31 | 18 | 13 |
| **Frailty Phenotype, n (%)** |  |  |  |
| Robust | 23,787 (65%) | 11,859 (63%) | 11,928 (67%) |
| Pre-frail | 12,178 (33%) | 6,545 (35%) | 5,633 (32%) |
| Frail | 464 (1.3%) | 315 (1.7%) | 149 (0.8%) |
| Missing | 260 | 163 | 97 |
| **Hypertension, n (%)** | 7,398 (20%) | 3,048 (16%) | 4,350 (24%) |
| **Ischaemic heart disease, n (%)** | 1,081 (2.9%) | 281 (1.5%) | 800 (4.5%) |
| **Stroke, n (%)** | 246 (0.7%) | 95 (0.5%) | 151 (0.8%) |
| **Diabetes, n (%)** | 985 (2.7%) | 320 (1.7%) | 665 (3.7%) |
| **CKD (G3-G5), n (%)** | 246 (0.7%) | 133 (0.7%) | 113 (0.6%) |
| **Cancer, n (%)** | 2,677 (7.3%) | 1,616 (8.6%) | 1,061 (6.0%) |
| **Thyroid disease, n (%)** | 1,736 (4.7%) | 1,440 (7.6%) | 296 (1.7%) |
| **COPD, n (%)** | 288 (0.8%) | 106 (0.6%) | 182 (1.0%) |
| *Body mass index (BMI), estimated glomerular filtration rate (eGFR), eGFR-creatinine (eGFRcr), eGFR-cystatin C (eGFRcys), eGFR-creatinine cystatin C (eGFRcr-cys), chronic kidney disease (CKD), chronic obstructive pulmonary disease (COPD* | | | |

Table S5: DEXA cohort – Participant characteristics (case control)

| **Characteristic** | **Overall**  N = 34,728 | **Female**  N = 17,803 | **Male**  N = 16,925 |
| --- | --- | --- | --- |
| **Age, Baseline visit (years), Median (IQR)** | 56 (49, 61) | 55 (48, 61) | 57 (50, 62) |
| **Ethnicity, n (%)** |  |  |  |
| White | 33,446 (96%) | 17,154 (96%) | 16,292 (96%) |
| Black | 264 (0.8%) | 143 (0.8%) | 121 (0.7%) |
| Asian | 440 (1.3%) | 162 (0.9%) | 278 (1.6%) |
| Mixed | 180 (0.5%) | 122 (0.7%) | 58 (0.3%) |
| Other | 310 (0.9%) | 181 (1.0%) | 129 (0.8%) |
| Unknown | 88 (0.3%) | 41 (0.2%) | 47 (0.3%) |
| **Smoking status, n (%)** |  |  |  |
| Current | 2,228 (6.4%) | 955 (5.4%) | 1,273 (7.5%) |
| Previous | 11,615 (33%) | 5,505 (31%) | 6,110 (36%) |
| Never | 20,814 (60%) | 11,307 (64%) | 9,507 (56%) |
| Prefer not to answer | 71 (0.2%) | 36 (0.2%) | 35 (0.2%) |
| **Height (cm), Median (IQR)** | 170 (163, 177) | 163 (159, 168) | 177 (172, 181) |
| **Weight (kg), Median (IQR)** | 76 (66, 86) | 68 (61, 76) | 83 (76, 92) |
| **BMI (kg/m^2^), Median (IQR)** | 26.0 (23.7, 28.9) | 25.2 (22.9, 28.5) | 26.7 (24.6, 29.2) |
| **Body surface area (m^2^), Median (IQR)** | 1.87 (1.72, 2.02) | 1.74 (1.64, 1.84) | 2.00 (1.90, 2.11) |
| **Diet, n (%)** |  |  |  |
| Omnivore | 33,923 (98%) | 17,272 (97%) | 16,651 (98%) |
| Vegetarian | 805 (2.3%) | 531 (3.0%) | 274 (1.6%) |
| **Handedness, n (%)** |  |  |  |
| Left-handed | 3,323 (9.6%) | 1,561 (8.8%) | 1,762 (10%) |
| Right-handed | 30,861 (89%) | 16,012 (90%) | 14,849 (88%) |
| Use both right and left hands equally | 541 (1.6%) | 229 (1.3%) | 312 (1.8%) |
| Prefer not to answer | 3 (<0.1%) | 1 (<0.1%) | 2 (<0.1%) |
| **Creatinine (mg/dL), Median (IQR)** | 0.81 (0.70, 0.92) | 0.71 (0.65, 0.79) | 0.91 (0.83, 1.00) |
| **Cystatin C (mg/L), Median (IQR)** | 0.86 (0.79, 0.95) | 0.83 (0.76, 0.91) | 0.90 (0.83, 0.98) |
| **eGFRcr, Median (IQR)** | 98 (88, 104) | 98 (88, 105) | 97 (88, 104) |
| **eGFRcys, Median (IQR)** | 93 (82, 103) | 96 (83, 104) | 91 (81, 102) |
| **eGFRcr-cys, Median (IQR)** | 99 (89, 107) | 100 (90, 108) | 97 (88, 106) |
| **Hospital Frailty Risk Score, Median, (Min - Max)** | 0.00 (0.00 - 16.60) | 0.00 (0.00 - 16.60) | 0.00 (0.00 - 12.60) |
| **Hospital Frailty Risk Score Level, n (%)** |  |  |  |
| High Risk | 1 (<0.1%) | 1 (<0.1%) | 0 (0%) |
| Intermediate Risk | 392 (1.1%) | 207 (1.2%) | 185 (1.1%) |
| Low Risk | 34,335 (99%) | 17,595 (99%) | 16,740 (99%) |
| **Charlson Comorbidity Index, Median, (Min - Max)** | 0 (0 - 9) | 0 (0 - 9) | 0 (0 - 9) |
| **Charlson Comorbidity Index Level, n (%)** |  |  |  |
| 0 | 32,979 (95%) | 16,837 (95%) | 16,142 (95%) |
| 1-2 | 1,580 (4.5%) | 857 (4.8%) | 723 (4.3%) |
| >3 | 169 (0.5%) | 109 (0.6%) | 60 (0.4%) |
| **Frailty Index, Median (IQR)** | 0.09 (0.06, 0.14) | 0.10 (0.06, 0.14) | 0.09 (0.06, 0.14) |
| **Frailty Phenotype, n (%)** |  |  |  |
| Robust | 22,751 (66%) | 11,319 (64%) | 11,432 (68%) |
| Pre-frail | 11,553 (33%) | 6,195 (35%) | 5,358 (32%) |
| Frail | 424 (1.2%) | 289 (1.6%) | 135 (0.8%) |
| **Hypertension, n (%)** | 6,932 (20%) | 2,846 (16%) | 4,086 (24%) |
| **Ischaemic heart disease, n (%)** | 993 (2.9%) | 258 (1.4%) | 735 (4.3%) |
| **Stroke, n (%)** | 222 (0.6%) | 83 (0.5%) | 139 (0.8%) |
| **Diabetes, n (%)** | 913 (2.6%) | 294 (1.7%) | 619 (3.7%) |
| **CKD (G3-G5), n (%)** | 221 (0.6%) | 118 (0.7%) | 103 (0.6%) |
| **Cancer, n (%)** | 2,521 (7.3%) | 1,515 (8.5%) | 1,006 (5.9%) |
| **Thyroid disease, n (%)** | 1,623 (4.7%) | 1,345 (7.6%) | 278 (1.6%) |
| **COPD, n (%)** | 260 (0.7%) | 91 (0.5%) | 169 (1.0%) |
| *Body mass index (BMI), estimated glomerular filtration rate (eGFR), eGFR-creatinine (eGFRcr), eGFR-cystatin C (eGFRcys), eGFR-creatinine cystatin C (eGFRcr-cys), chronic kidney disease (CKD), chronic obstructive pulmonary disease (COPD* | | | |

**2. TTFMV and CMI by age category**

**Table S6: Median TTFMV and CMI by age category**

|  | **Median TTFMV (L/1.73m^2^)** | | **Median CMI (mg/day/1.73m^2^)** | |
| --- | --- | --- | --- | --- |
| **Age category (years)** | Male | Female | Male | Female |
| 38-43 | 11.3 | 8.56 | 1314 | 1069 |
| 44-48 | 11.1 | 8.38 | 1290 | 1046 |
| 49-53 | 10.9 | 8.17 | 1241 | 987 |
| 54-58 | 10.7 | 8.05 | 1187 | 935 |
| 59-63 | 10.4 | 7.94 | 1131 | 890 |
| 64-68 | 10.1 | 7.83 | 1078 | 852 |
| 69-73 | 9.86 | 7.78 | 1045 | 824 |

**3. Outcome variables by cohort**

**Table S7: Outcome variables by cohort**

| **Outcome** | **Whole cohort**^1^ | | **MRI Cohort**^1^ | | **DXA Cohort**^1^ | |
| --- | --- | --- | --- | --- | --- | --- |
|  | **Female**,  N = 244,260 | **Male**,  N = 206,552 | **Female**,  N = 17,611 | **Male**,  N = 16,188 | **Female**,  N = 17,803 | **Male**,  N = 16,925 |
| **CMI (mg/day/1.73m^2^)** | 907 (798, 1,025) | 1,138 (1,006, 1,281) | 956 (854, 1,065) | 1,185 (1,062, 1,314) | 956 (852, 1,066) | 1,183 (1,058, 1,315) |
| **Hand grip strength (kg/m^2^)**** | 9.3 (7.80, 10.90) | 13.2 (11.40, 15.00) | 9.8 (8.2, 11.3) | 13.6 (11.9, 15.4) | 9.8 (8.20, 11.30) | 13.6 (11.90, 15.40) |
| **TTFMV (L/1.73m^2^)*** |  |  | 8.11 (7.60, 8.66) | 10.62 (9.95, 11.33) |  |  |
| **DEXA-ALM (kg/1.73m^2^)*** |  |  |  |  | 17.15 (16.25, 18.13) | 21.88 (20.72, 23.08) |
| *Normalised to standardised BSA, **Normalised to height^2^  ^1^Median (IQR)  *Creatinine muscle index (CMI), total thigh fat-free muscle volume (TTFMV), dual energy X-ray absorptiometry appendicular lean mass (DEXA-ALM)* | | | | | | |

**4. Regression models using anterior and posterior muscle compartment volumes**

**Table S8: Regression models using anterior and posterior muscle compartment volumes**

| **Outcome variable: Anterior thigh fat free muscle volume (L/1.73m^2^)** | | | |
| --- | --- | --- | --- |
| *Model* | *Coefficient (SE, p)* | *R (95% CI)* | *R^2^ (95% CI)* |
| Unweighted linear regression: MRI cohort (males and females) | 0.002 (1.27e-5, p<0.001) | 0.62 (0.61-0.62) | 0.38 (0.37-0.39) |
| Unweighted linear regression: Males (MRI) | 0.001 (1.76e-5, p<0.001) | 0.38 (0.37-0.39) | 0.14 (0.13-0.16) |
| Unweighted linear regression: Females (MRI) | 0.001 (1.49e-5, p<0.001) | 0.38 (0.37-0.40) | 0.15 (0.14-0.16) |
| Weighted linear regression: Males (MRI) | 0.003 (2.54e-5, p<0.001) | 0.69 (0.63-0.75) | 0.48 (0.40-0.55) |
| Weighted linear regression: Females (MRI) | 0.003 (2.49e-5, p<0.001) | 0.64 (0.57-0.70) | 0.41 (0.34-0.50) |
| **Outcome variable: Posterior thigh fat free muscle volume (L/1.73m^2^)** | | | |
| Unweighted linear regression: MRI cohort (males and females) | 0.003 (2.00e-5, p<0.001) | 0.59 (0.58-0.60) | 0.35 (0.34-0.36) |
| Unweighted linear regression: Males (MRI) | 0.001 (2.52e-5, p<0.001) | 0.31 (0.30-0.33) | 0.10 (0.09-0.11) |
| Unweighted linear regression: Females (MRI) | 0.001 (2.31e-5, p<0.001) | 0.31 (0.30-0.33) | 0.10 (0.09-0.11) |
| Weighted linear regression: Males (MRI) | 0.004 (3.83e-5, p<0.001) | 0.63 (0.55-0.71) | 0.40 (0.30-0.48) |
| Weighted linear regression: Females (MRI) | 0.004 (4.11e-5, p<0.001) | 0.59 (0.52-0.67) | 0.35 (0.25-0.46) |

**5. Sensitivity analysis: Ethnicity, Age and BMI**

Figure S1: Regression lines split by ethnicity


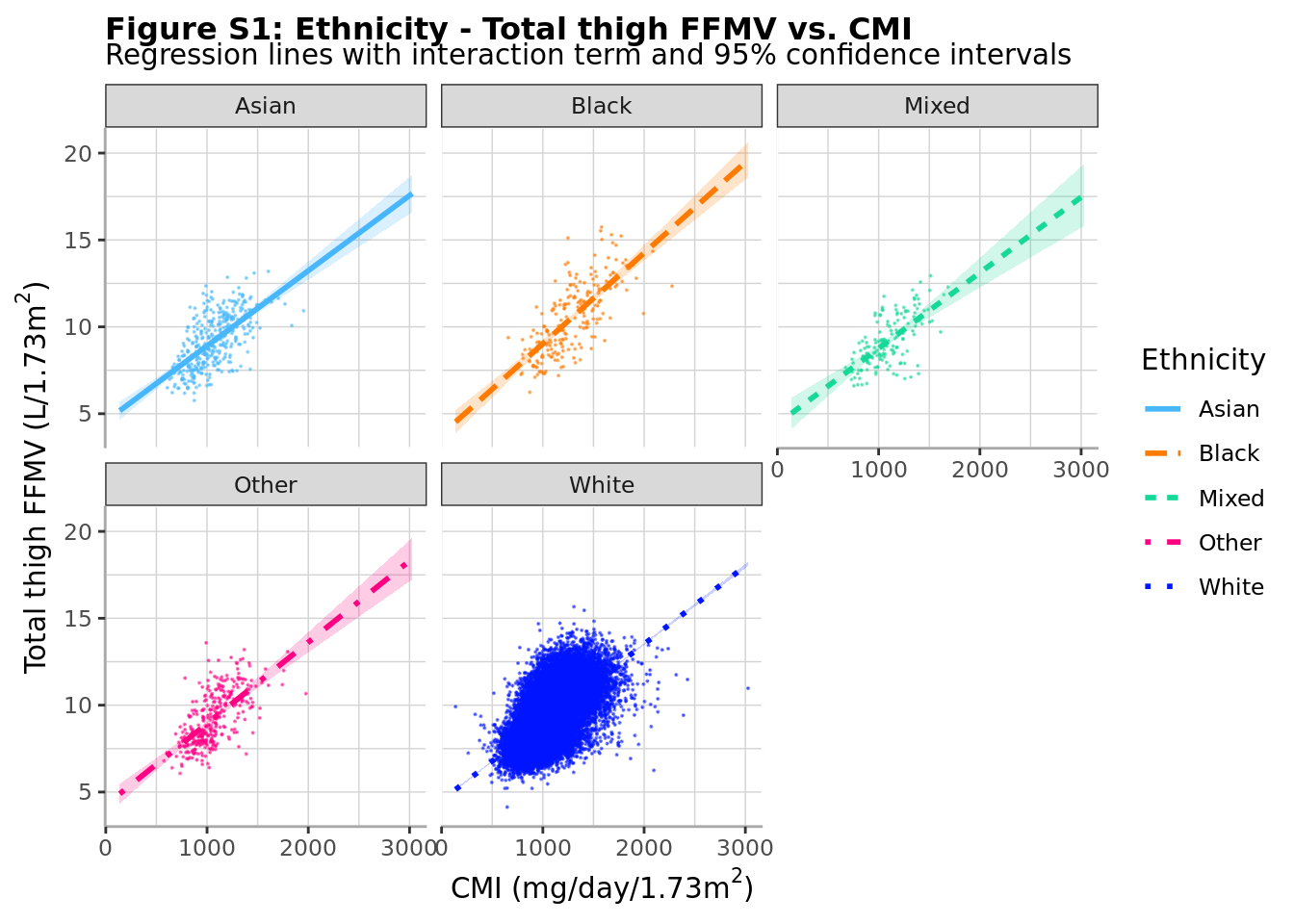


Figure S2: Regression lines split by age


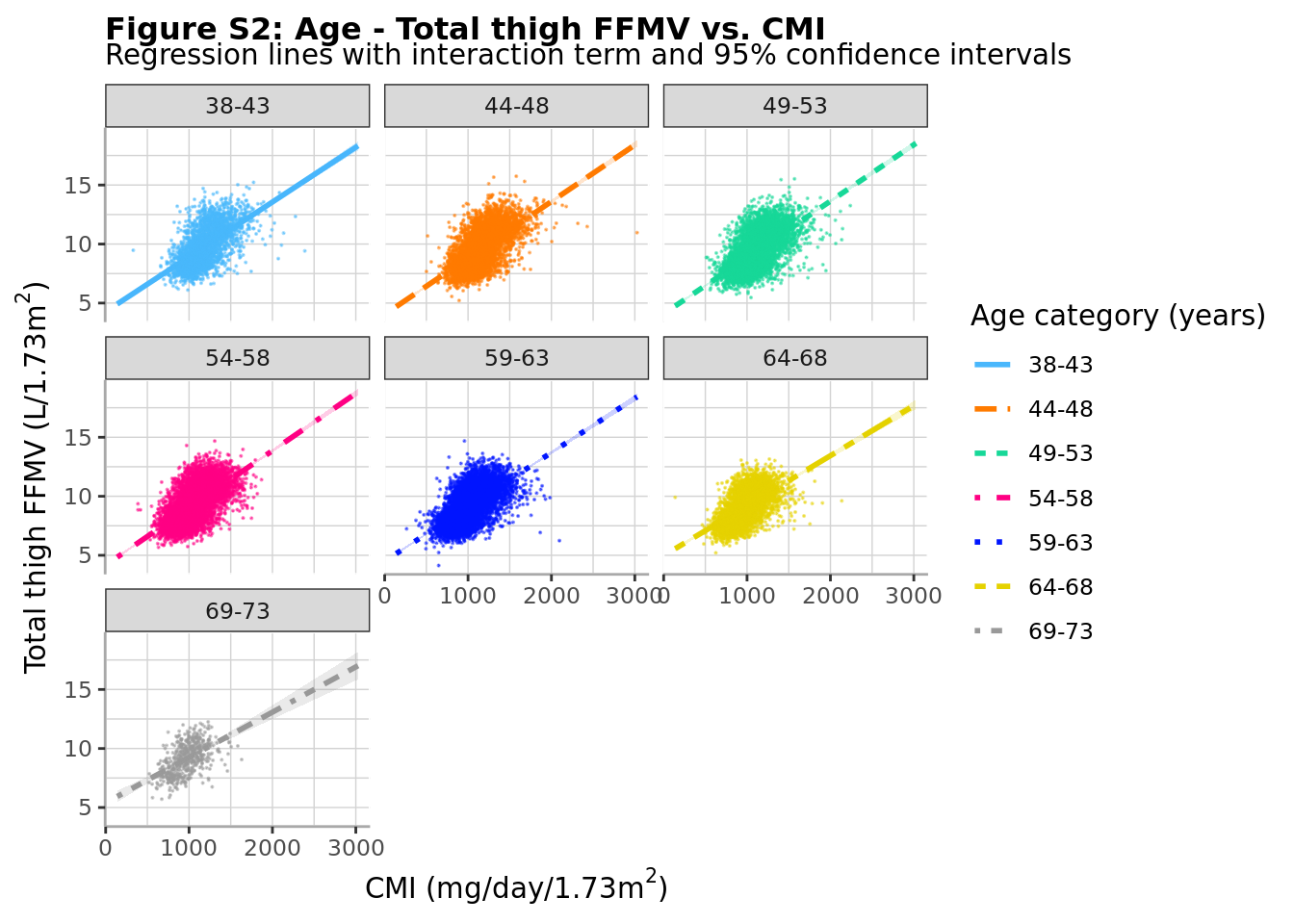


Figure S3: Regression lines split by BMI category


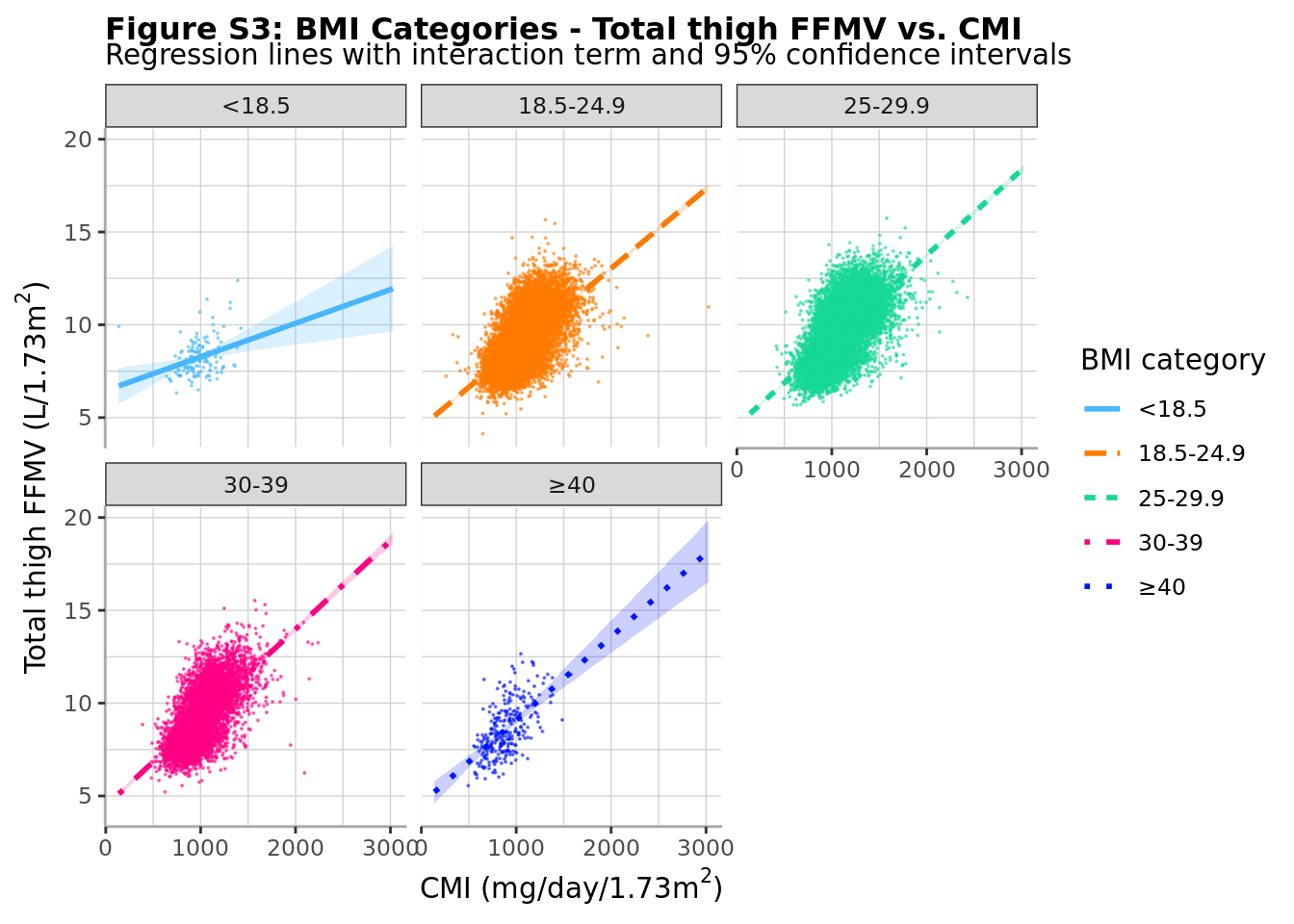


**6. Unweighted and weighted regression modelling in the DEXA cohort**

**Table S9: Unweighted and weighted regression modelling in the DXA cohort using DEXA-ALM**

|  | **Outcome variable: DEXA-ALM (kg/1.73m^2^)** | | |
| --- | --- | --- | --- |
| *Model* | *Coefficient (SE, p)* | *R (95% CI)* | *R^2^ (95% CI)* |
| Unweighted linear regression: DEXA cohort | 0.007 (5.85e-05, p<0.001) | 0.56 (0.56-0.57) | 0.32 (0.31-0.33) |
| Unweighted linear regression: Males | 0.003 (6.68e-05, p<0.001) | 0.28 (0.22-0.24) | 0.08 (0.07-0.09) |
| Unweighted linear regression: Females | 0.002 (6.34e-05, p<0.001) | 0.23 (0.22-0.24) | 0.05 (0.05-0.06) |
| Weighted linear regression: Males | 0.010 (1.38e-4, p<0.001) | 0.48 (0.26-0.66) | 0.23 (0.06-0.42) |
| Weighted linear regression: Females | 0.010 (1.40e-4, p<0.001) | 0.49 (0.39-0.58) | 0.24 (0.14-0.33) |

**7. ROC curves for TTFFMV ≤2 SD and ≤2.5 SD below the mean using CMI**

Figure S4: ROC curve for detection of total thigh fat-free muscle volume (TTFMV) ≤2 SD below the mean using creatinine muscle index (CMI) in males (a) ROC curve for detection of TTFMV ≤2 SD below the mean using CMI in females (b). ROC curve for detection of TTFMV ≤2.5 SD below the mean using CMI in males (c) ROC curve for detection of TTFMV ≤2.5 SD below the mean using CMI in females (d)


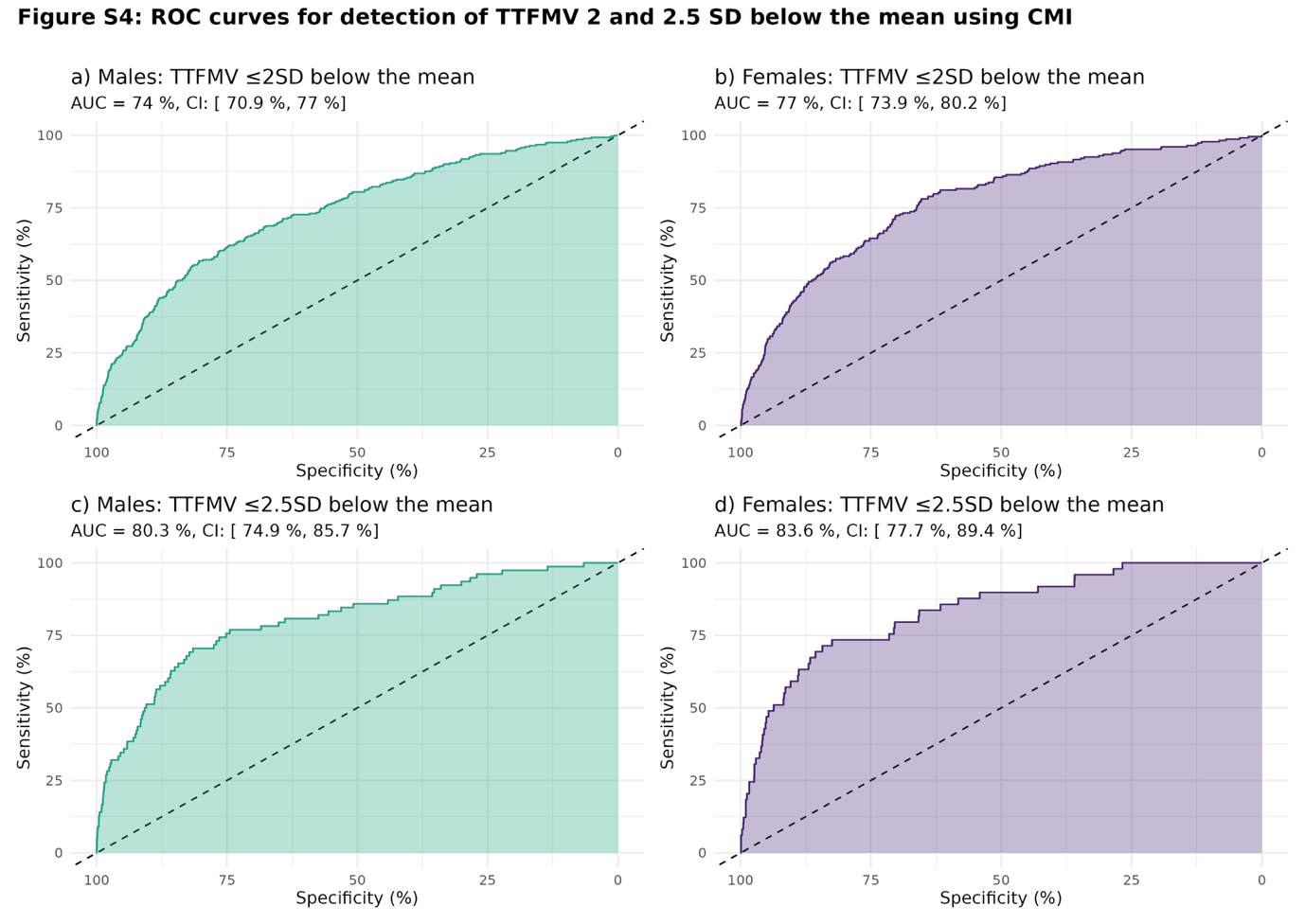


**8. ROC curves for sarcopenia detection as per European Working Group on Sarcopenia in Older People (EWGSOP) guideline^1^ definitions using CMI**

Figure S5: Graph showing ROC curves for the detection of confirmed and probable sarcopenia using CMI. Conformed sarcopenia was defined using the EWSGSOP definitions for males (a): hand grip strength <27kg and appendicular skeletal muscle/height^2^ <7kg/m^2^, and females (b): hand grip strength <16kg and appendicular skeletal muscle/height^2^ <5.5kg/m^2^. Probable sarcopenia was defined using the EWSGSOP definitions for males (c): hand grip strength <27kg, and females (d): hand grip strength <16kg.


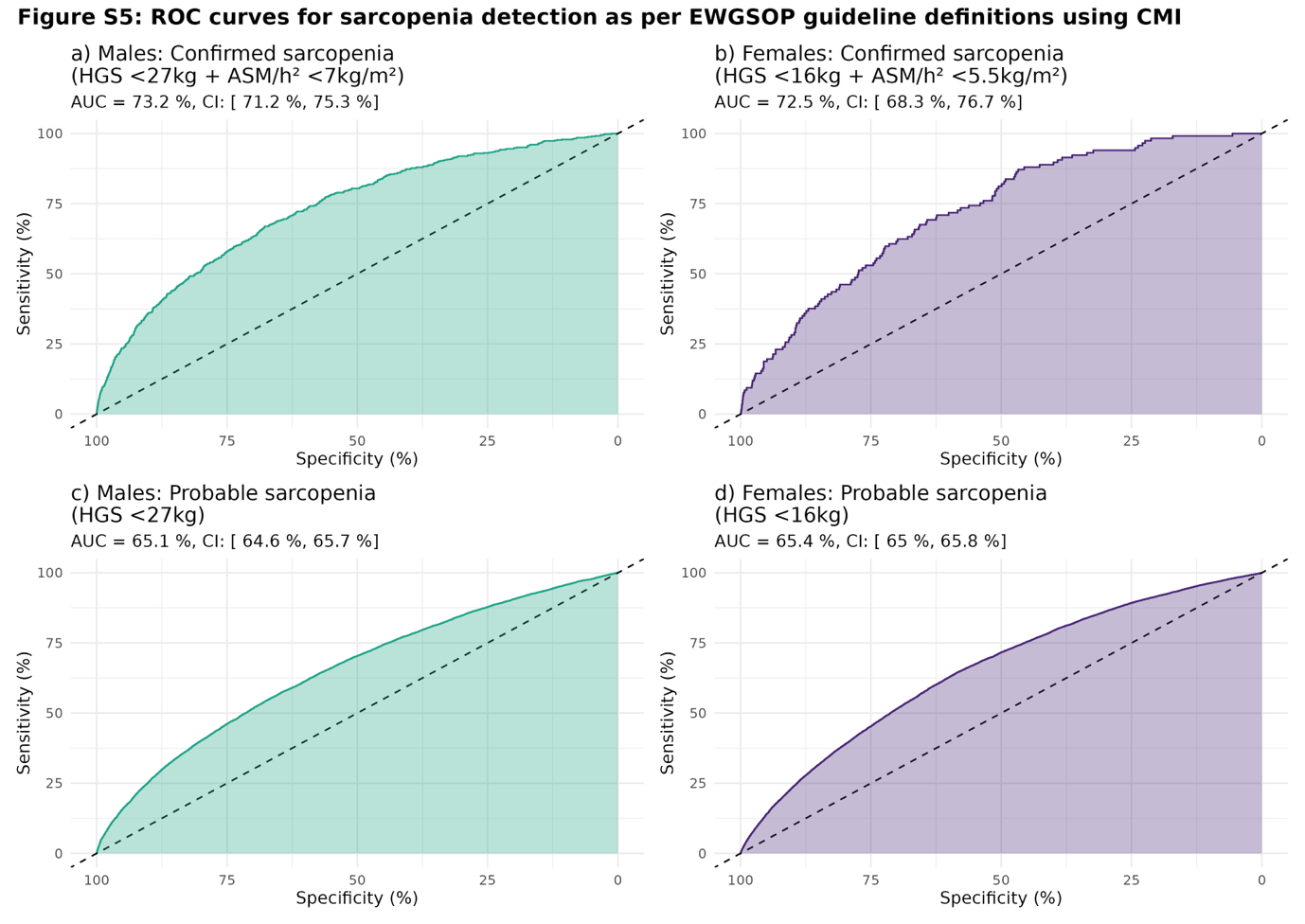


**9. Shoenfield residuals plots**

Figure S6: Scaled Shoenfield residuals plots for cox-proportional hazards models: a) males, b) females

Scaled Schoenfield residual plots are used to assess whether covariate effects remain constant over time in a Cox proportional hazards model. Scaled plots adjust for the variance of estimated coefficients. If the proportional hazard assumption holds the smoothed residuals fluctuate randomly around zero, if it fails they show drift over time. The plots below show that the effects of CMI (our covariate of interest) shows modest attenuation over time, however the change in magnitude is limited and the direction of the association remains consistently positive over time. Due to the very large sample size this drift is statistically significant, however it is unlikely to be clinically important over the time period examined in the study. The hazard ratios provided reflect the mean hazard over the follow-up period.

**
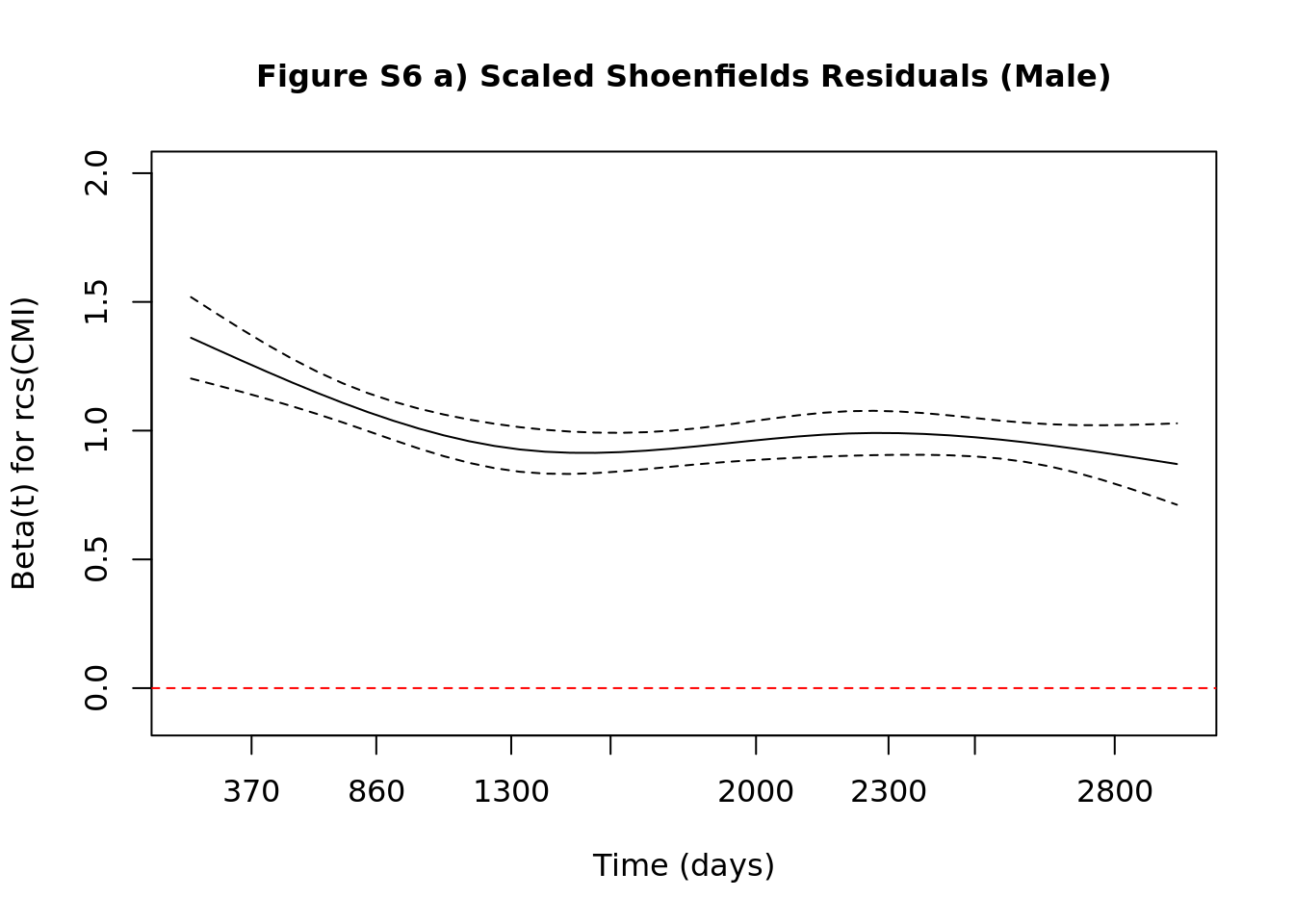
**

**
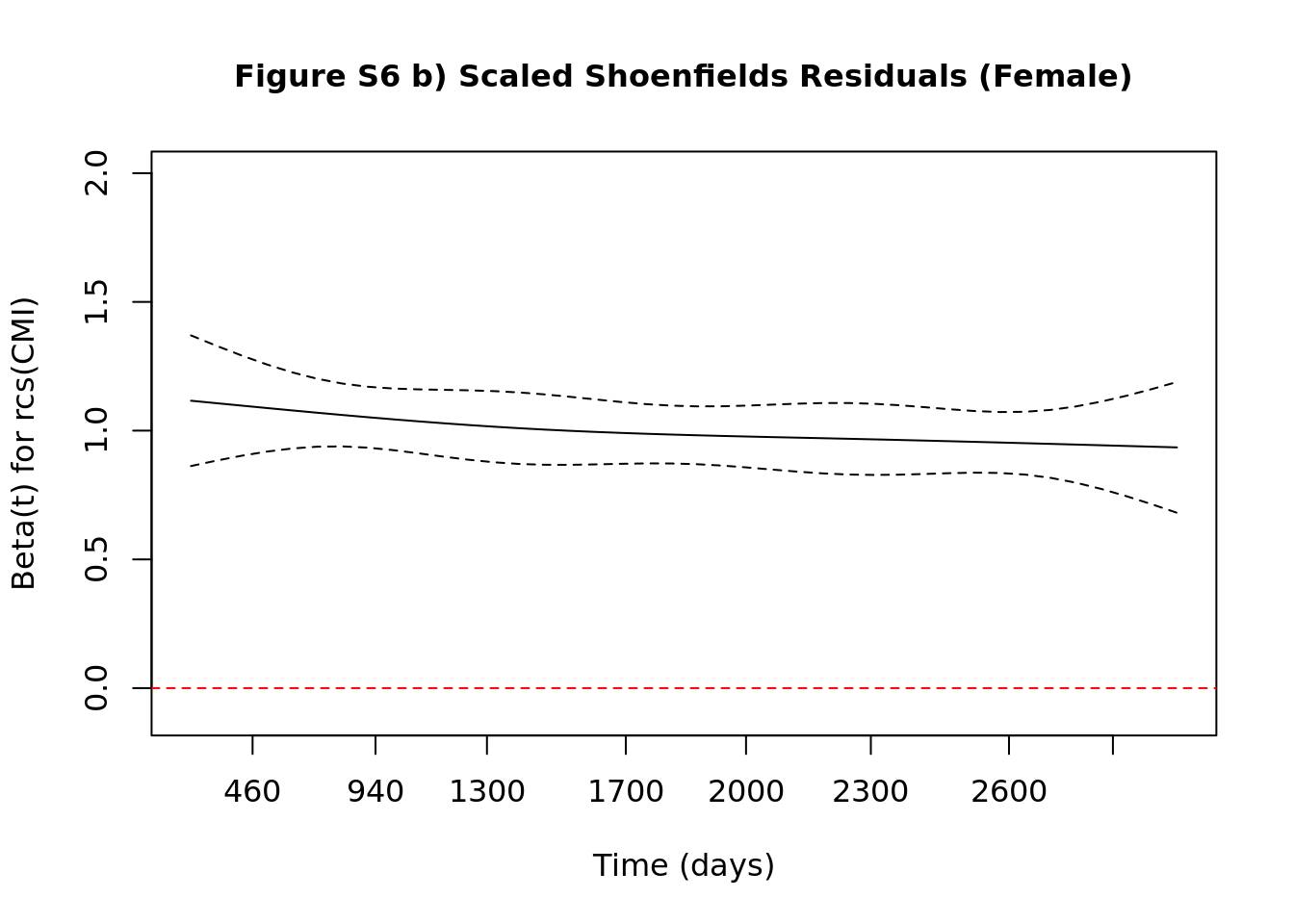
**

**10. Cohort selection process**

Figure S7: Cohort selection process


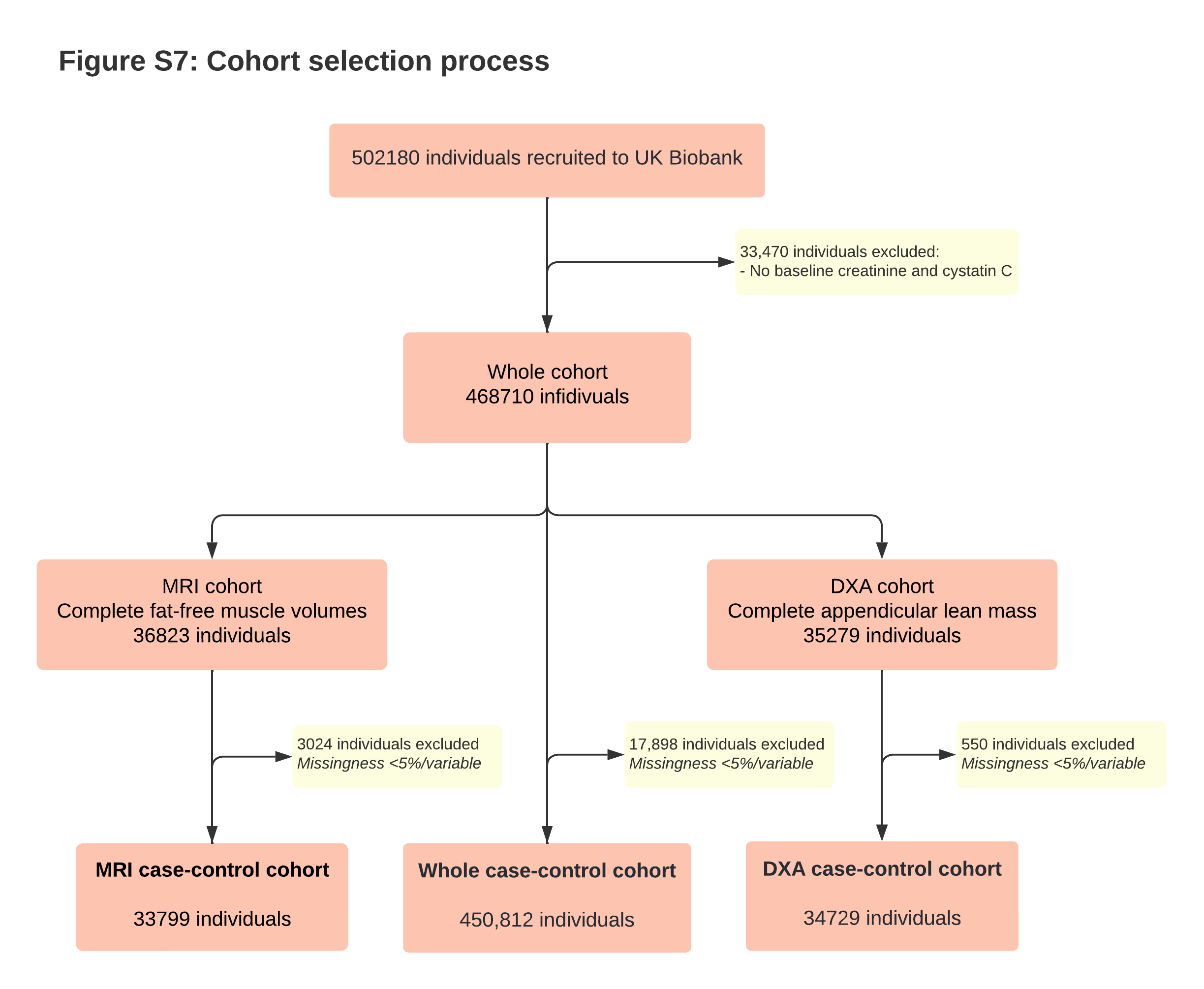


**11. Detailed variable definitions**

*Body Surface Area*

Body surface area was calculated using the Du Bois formula^2^ using baseline variables for height and weight.

*Ethnicity*

Ethnicity was re-coded as per the Office of National Statistics in the following groups (<https://www.ons.gov.uk/peoplepopulationandcommunity/culturalidentity/ethnicity/methodologies/ethnicgroupnationalidentitylanguageandreligionqualityinformationforcensus2021>).

- White: White, British, Irish, Any other white background
- Asian or Asian British: Indian, Pakistani, Bangladeshi, Any other Asian background, Asian or Asian British
- Black or Black British: Caribbean, African, Any other Black background
- Mixed: White and Black Caribbean, White and Black African, White and Asian, Any other mixed background, Mixed
- Other: Chinese, Other ethnic group
- Unknown: Prefer not to answer, Do not know

*Fat free muscle volume*

The fat-free volume is defined as ‘the volume of all voxels with a fat fraction <50%’, this allows the muscle and fat volumes to be separated, giving a volume for viable muscle. Fat-free muscle volume of the anterior and posterior thigh compartments was calculated from abdominal MRI scans using the AMRA (Advanced MR Analytics AB, AMRA, Sweden) Profiler system. Full methods of the MRI protocol (<https://biobank.ndph.ox.ac.uk/showcase/ukb/docs/body_mri_explan.pdf>, <https://biobank.ndph.ox.ac.uk/showcase/ukb/docs/Abdominal_scan_protocol.pdf>) and body composition profiling (<https://biobank.ndph.ox.ac.uk/ukb/ukb/docs/AMRA_derived_explan_doc.pdf>) used in UK Biobank have been published.

Anterior thigh muscles include quadriceps femoris, sartorius and tensor fascia lata. Posterior thigh muscles include gluteus, iliacus, adductor and hamstring muscles.

*DXA appendicular lean mass (DXA ALM)*

DXA ALM was calculated using the combined appendicular lean mass values (bilateral arm lean mass + bilateral leg lean mass). Images were acquired using the GE-Lunar-iDXA machine and a full protocol has been published by UK Biobank (<https://biobank.ndph.ox.ac.uk/showcase/ukb/docs/DXA_explan_doc.pdf>). Values were normalised to standardised body surface area for the regression analysis against CMI (kg/1.73m^2^).

*Hand grip strength*

Hand-grip strength was measured at the baseline assessment (2006-2010) using a Jamar J00105 hydraulic hand dynamometer.^4^ The units of measurement are in kg. The dominant hand grip strength for each participant was used, if they were ambidextrous or preferred not to answer the value corresponding to the right hand was used. Values were normalised to height squared (kg/m^2^) for the regression analysis.

*Serum creatinine and cystatin C*

Blood values used were measured at baseline; quality procedure protocols of biochemical assays have been described.^5^ Serum creatinine was measured using enzymatic analysis on a Beckman Coulter AU5800. Serum cystatin C was measured by latex enhanced immunoturbidimetric analysis on a Siemens ADVIA 1800.

*eGFR_cys_ and eGFR*

eGFRcys was calculated using the 2012 CKD-EPI cystatin C equation and eGFRcr using the 2021 CKD-EPI creatinine equation (race-free).^6,7^

*Charlson Comorbidity Index*

The Charlson Comorbidity Index (CCI) is a commonly used index to classify comorbid conditions; identifying those associated with a risk for mortality.^8^Diagnoses made prior to recruitment date were included. Participants were grouped into those with a CCI score of 0, 1-2 or ≥3. A higher score is associated with a higher risk of mortality. Conditions were identified using the methods described in the comorbidities section. Mapping was completed using ICD-10 Coding and using the R package: Comorbidity.^9^ The updated mapping and weighting by Quan was used.^10,11^

*Hospital Frailty Risk Score*

The Hospital Frailty Risk Score (HFRS) uses ICD-10 diagnostic codes to identify frailty. Diagnoses made prior to recruitment date were included. Participants were divided into low (≤4), medium (5-15) and high (≥16) risk for frailty. Mapping was performed using ICD-10 coding with weighting described by Gilbert et al.^12^

*UK Biobank Frailty Index (UKBFI)*

The UKBFI validated by Williams et al.^13^ has been used to identify the presence of frailty within the study population. It is a frailty index derived from 49 data items specific to UK Biobank data, with higher values indicating a higher level of frailty. Variables are composed of self-reported data; the full coding can be found in the supplement. In brief, variables are coded absent or present, the total score of variables present is divided by the total variables available for each participant. Items are discounted if they are not available, if more than 10 items are not available the index cannot be calculated.

*Fried Frailty phenotype*

The frailty phenotypes; not frail, pre-frail and frail, originally described by Fried et al.^14^ have been adapted for use in the UK Biobank.^15^ Frailty phenotype is calculated using 4 self-reported variables and hand grip strength.

*Sarcopenia*

Sarcopenia was defined using the European Working Group on Sarcopenia in Older People (EWGSOP2) criteria^1^:

- Confirmed sarcopenia: Males (a): hand grip strength <27kg and appendicular skeletal muscle/height^2^ <7kg/m^2^, and Females (b): hand grip strength <16kg and appendicular skeletal muscle/height^2^ <5.5kg/m^2^.
- Probable sarcopenia: Males (c): hand grip strength <27kg, and Females (d): hand grip strength <16kg.

**12. Comorbidity coding**

Diagnoses made prior to date of recruitment to UK Biobank for each individual. The coding for self-reported illness can be found here for non-cancer illness: <https://biobank.ndph.ox.ac.uk/showcase/coding.cgi?id=6> and cancer: <https://biobank.ndph.ox.ac.uk/showcase/coding.cgi?id=3>.

| **Source** | **Associated coding** |
| --- | --- |
| **Hypertension** | |
| Self-reported illness data | 1065, 1072 |
| ICD-10 | I10, I11, I110, I119, I12, I120, I129, I13, I130, I131, I132, I139, I15, I150, I151, I152, I158, I159, I674 |
| ICD-9 | 401, 4010, 4011, 4019, 402, 4020, 4021, 4029, 403, 4030, 4031, 4039, 404, 4040, 4041, 4049, 405, 4050, 4051, 4059, 4372 |
| **Ischaemic Heart Disease** | |
| UK Biobank algorithmic defined outcomes: Ischaemic heart disease (MI, STEMI, NSTEMI) | |
| Self-reported illness data | IHD (inc. angina): 1074, 1075 |
| ICD-10 | I20, I200, I201, I208, I209, I21, I210, I211, I212, I213, I214, I219, I22, I220, I221, I228, I229, I23, I230, I231, I232, I233, I234, I235, I236, I238, I241, I248, I249, I25, I251, I252, I253, I254, I255, I256, I258, I259 |
| ICD-9 | 410, 4101, 4102, 4103, 4104, 4105, 4106, 4107, 4108, 4109, 411, 412, 42979, 413, 4140, 4141, 4149, 414, 4148 |
| **Stroke** | |
| UK Biobank algorithmic defined outcomes: Stroke | |
| Self-reported illness data | 1081, 1086, 1491, 1583 |
| ICD-10 | I60, I600, I601, I602, I603, I604, I605, I606, I607, I608, I609, I61, I610, I611, I612, I613, I614, I615, I616, I618, I619, I63, I630, I631, I632, I633, I634, I635, I636, I638, I639, I64X, I69, I690, I691, I692, I693, I694, I698 |
| ICD-9 | 430A, 430, 431, 4333, 4339, 4331, 4332, 4338, 4330, 4340, 4341, 4349, 436, 438 |
| **Diabetes** | |
| Self-reported illness data | 1220, 1222, 1223 |
| ICD-10 | E10, E100, E101, E102, E103, E104, E105, E106, E107, E108, E109, E11, E110, E111, E112, E113, E114, E115, E116, E117, E118, E119, E13, E130, E131, E132, E133, E134, E135, E136, E137, E138, E139, E14, E140, E141, E142, E143, E144, E145, E146, E147, E148, E149, G590, G632, H280, H360, M142, N083, O340, O241, O243 |
| ICD-9 | 250, 2500, 2501,2502, 2503,2504, 2505, 2506, 2507, 2509, 3572A |
| **Chronic Kidney Disease (G3-G5)** | |
| eGFR based on admission creatinine | ≤59ml/min/1.73m^2^ |
| **Cancer** | |
| Self-reported illness data | 1001, 1002, 1003, 1004, 1005, 1006, 1007, 1008, 1009, 1010, 1011, 1012, 1015, 1016, 1017, 1018, 1019, 1020, 1021, 1022, 1023, 1024, 1025, 1026, 1027, 1028, 1029, 1030, 1031, 1032, 1033, 1034, 1035, 1036, 1037, 1038, 1039, 1040, 1041, 1042, 1043, 1044, 1045, 1046, 1047, 1048, 1050, 1051, 1052, 1053, 1055, 1056, 1058, 1059, 1060, 1061, 1062, 1063, 1064, 1065, 1067, 1068, 1070, 1071, 1072, 1073, 1074, 1075, 1076, 1077, 1078, 1079, 1080, 1081, 1082, 1084, 1085, 1086, 1087, 1088, 99999 |
| ICD-10 | C00, C000, C001, C002, C003, C004, C005, C006, C008, C009, C01X, C02, C020, C021, C022, C023, C024, C028, C029, C03, C030, C031, C039, C04, C040, C041, C048, C049, C05, C050, C051, C052, C058, C059, C06, C060, C061, C062, C068, C069, C07X, C08, C080, C081, C088, C089, C09, C090, C091, C098, C099, C10, C100, C101, C102, C103, C104, C108, C109, C11, C110, C111, C112, C113, C118, C119, C12X, C13, C130, C131, C132, C138, C139, C14, C140, C142, C148, C15, C150, C151, C152, C153, C154, C155, C158, C159, C16, C160, C161, C162, C163, C164, C165, C166, C168, C169, C17, C170, C171, C172, C173, C178, C179, C18, C180, C181, C182, C183, C184, C185, C186, C187, C188, C189, C19X, C20X, C21, C210, C211, C212, C218, C22, C220, C221, C222, C223, C224, C227, C229, C23X, C24, C240, C241, C248, C249, C25, C250, C251, C252, C253, C254, C257, C258, C259, C26, C260, C261, C268, C269, C30, C300, C301, C31, C310, C311, C312, C313, C318, C319, C32, C320, C321, C322, C323, C328, C329, C33X, C34, C340, C341, C342, C343, C348, C349, C37X, C38, C380, C381, C382, C383, C384, C388, C39, C390, C398, C399, C40, C400, C401, C402, C403, C408, C409, C41, C410, C411, C412, C413, C414, C418, C419, C43, C430, C431, C432, C433, C434, C435, C436, C437, C438, C439, C44, C440, C441, C442, C443, C444, C445, C446, C447, C448, C449, C45, C450, C451, C452, C457, C459, C46, C460, C461, C462, C463, C467, C468, C469, C47, C470, C471, C472, C473, C474, C475, C476, C478, C479, C48, C480, C481, C482, C488, C49, C490, C491, C492, C493, C494, C495, C496, C498, C499, C50, C500, C501, C502, C503, C504, C505, C506, C508, C509, C51, C510, C511, C512, C518, C519, C52X, C53, C530, C531, C538, C539, C54, C540, C541, C542, C543, C548, C549, C55X, C56X, C57, C570, C571, C572, C573, C574, C577, C578, C579, C58X, C60, C600, C601, C602, C608, C609, C61X, C62, C620, C621, C629, C63, C630, C631, C632, C637, C638, C639, C64X, C65X, C66X, C67, C670, C671, C672, C673, C674, C675, C676, C677, C678, C679, C68, C680, C681, C688, C689, C69, C690, C691, C692, C693, C694, C695, C696, C698, C699, C70, C700, C701, C709, C71, C710, C711, C712, C713, C714, C715, C716, C717, C718, C719, C72, C720, C721, C722, C723, C724, C725, C728, C729, C73X, C74, C740, C741, C749, C75, C750, C751, C752, C753, C754, C755, C758, C759, C76, C760, C761, C762, C763, C764, C765, C767, C768, C77, C770, C771, C772, C773, C774, C775, C778, C779, C78, C780, C781, C782, C783, C784, C785, C786, C787, C788, C79, C790, C791, C792, C793, C794, C795, C796, C797, C798, C799, C80, C800, C809, C81, C810, C811, C812, C813, C814, C817, C819, C82, C820, C821, C822, C823, C824, C825, C826, C827, C829, C83, C830, C831, C833, C835, C837, C838, C839, C84, C840, C841, C844, C845, C846, C847, C848, C849, C85, C851, C852, C857, C859, C86, C860, C861, C862, C863, C864, C865, C866, C88, C880, C882, C883, C884, C887, C889, C90, C900, C901, C902, C903, C91, C910, C911, C913, C914, C915, C916, C917, C918, C919, C92, C920, C921, C922, C923, C924, C925, C926, C927, C928, C929, C93, C930, C931, C933, C937, C939, C94, C940, C942, C943, C944, C946, C947, C95, C950, C951, C957, C959, C96, C960, C962, C964, C965, C966, C967, C968, C969, C97X |
| ICD-9 | 140, 1400, 1401, 1403, 1404, 1405, 1406, 1408, 1409, 141, 1410, 1411, 1412, 1413, 1414, 1415, 1416, 1418, 1419, 142, 1420, 1421, 1422, 1428, 1429, 143, 1430, 1431, 1438, 1439, 144, 1440, 1441, 1448, 1449, 145, 1450, 1451, 1452, 1453, 1454, 1455, 1456, 1458, 1459, 146, 1460, 1461, 1462, 1463, 1464, 1465, 1466, 1467, 1468, 1469, 147, 1470, 1471, 1472, 1473, 1478, 1479, 148, 1480, 1481, 1482, 1483, 1488, 1489, 149, 1490, 1491, 1498, 1499, 150, 1500, 1501, 1502, 1503, 1504, 1505, 1508, 1509, 151, 1510, 1511, 1512, 1513, 1514, 1515, 1516, 1518, 1519, 152, 1520, 1521, 1522, 1523, 1528, 1529, 153, 1530, 1531, 1532, 1533, 1534, 1535, 1536, 1537, 1538, 1539, 154, 1540, 1541, 1542, 1543, 1548, 155, 1550, 1551, 1552, 156, 1560, 1561, 1562, 1568, 1569, 157, 1570, 1571, 1572, 1573, 1574, 1578, 1579, 158, 1580, 1588, 1589, 159, 1590, 1591, 1598, 1599, 160, 1600, 1601, 1602, 1603, 1604, 1605, 1608, 1609, 161, 1610, 1611, 1612, 1613, 1618, 1619, 162, 1620, 1622, 1623, 1624, 1625, 1628, 1629, 163, 1630, 1631, 1638, 1639, 164, 1640, 1641, 1642, 1643, 1648, 1649, 165, 1650, 1658, 1659, 170, 1700, 1701, 1702, 1703, 1704, 1705, 1706, 1707, 1708, 1709, 171, 1710, 1712, 1713, 1714, 1715, 1716, 1717, 1718, 1719, 172, 1720, 1721, 1722, 1723, 1724, 1725, 1726, 1727, 1728, 1729, 173, 1730, 1731, 1732, 1733, 1734, 1735, 1736, 1737, 1738, 1739, 174, 1740, 1741, 1742, 1743, 1744, 1745, 1746, 1748, 1749, 175, 179, 180, 1800, 1801, 1808, 1809, 181, 182, 1820, 1821, 1828, 183, 1830, 1832, 1833, 1834, 1835, 1838, 1839, 184, 1840, 1841, 1842, 1843, 1844, 1848, 1849, 185, 186, 1860, 1869, 187, 1871, 1872, 1873, 1874, 1875, 1876, 1877, 1878, 1879, 188, 1880, 1881, 1882, 1883, 1884, 1885, 1886, 1887, 1888, 1889, 189, 1890, 1891, 1892, 1893, 1894, 1898, 1899, 190, 1900, 1901, 1902, 1903, 1904, 1905, 1906, 1907, 1908, 1909, 191, 1910, 1911, 1912, 1913, 1914, 1915, 1916, 1917, 1918, 1919, 192, 1920, 1921, 1922, 1923, 1928, 1929, 193, 194, 1940, 1941, 1943, 1944, 1945, 1946, 1948, 1949, 195, 1950, 1951, 1952, 1953, 1954, 1955, 1958, 196, 1960, 1961, 1962, 1963, 1965, 1966, 1968, 1969, 197, 1970, 1971, 1972, 1973, 1974, 1975, 1976, 1977, 1978, 198, 1980, 1981, 1982, 1983, 1984, 1985, 1986, 1987, 1988, 199, 1990, 1991, 200, 2000, 2001, 2002, 2008, 201, 2010, 2011, 2012, 2014, 2015, 2016, 2017, 2019, 202, 2020, 2021, 2022, 2023, 2024, 2025, 2026, 2028, 2029, 203, 2030, 2031, 2038, 204, 2040, 2041, 2042, 2048, 2049, 205, 2050, 2051, 2052, 2053, 2058, 2059, 206, 2060, 2061, 2062, 2068, 2069, 207, 2070, 2071, 2072, 2078, 208, 2080, 2081, 2082, 2088, 2089 |
| **Thyroid disease** | |
| Self-reported illness data | 1224, 1225, 1226, 1428 |
| ICD-10 | E01, E018, E02X, E03, E032, E033, E034, E035, E038, E039, E05, E050, E051, E052, E053, E054, E055, E058, E059, E06, E060, E061, E062, E063, E064, E065, E069, E07, E078, E079, E890, O905 |
| ICD-9 | 242, 2420, 2421, 2422, 2423, 2424, 2428, 2429, 244, 2440, 2441, 2442, 2443, 2448, 2449, 245, 2450, 2451, 2452, 2453, 2454, 2458, 2459, 246, 6481 |
| **COPD** | |
| UK Biobank algorithmic defined outcomes: COPD | |
| Self-reported illness data | 1112, 1113, 1472 |
| ICD-10 | J40, J41, J410, J411, J418, J42X, J43, J430, J431, J432, J438, J439, J44, J440, J441, J448, J449 |
| ICD-9 | 490, 4910, 4911, 4912, 49121, 49122, 4918, 4919, 492, 496 |

**13. Approach to linear regression analysis**

We sought to assess CMI as an index of muscle mass against two gold standards; TTFMV and DXA, measured in their respective subgroups of the UK Biobank population. CMI was compared with normalised TTFMV or DXA using linear regression with CMI as the explanatory variable. We considered both the relationships for male and female sex separately and in combination. As the UK Biobank recruited a predominantly healthy population, with relatively narrow age range and low ethnic diversity, normalised muscle mass observations were tightly clustered particularly after normalisation for size. This resulted in a large proportion of the variation between participants muscle mass or CMI being accounted for by measurement variation, rather than a biological difference in muscle. As measurement errors will not be correlated between different tests clustering will tend to flatten the regression line and reduce the correlation coefficient even if there is a strong relationship between biomarkers and the biological variable in question. Furthermore, in clinical practice we are interested in identifying individuals whose muscle mass lies significantly in a pathological range which, while present within the UK Biobank dataset, is outweighed by a preponderance of individuals clustered around the median.

Several methods exist to deal with imbalanced datasets in regression problems. A common approach in classification modelling; synthetic minority over-sampling technique (SMOTE) involves over-sampling or imputation of underrepresented data points and under-sampling of over-represented data.^16^ An alternative, which uses the full dataset without deletion or imputation, is to use weighted linear regression to increase the influence of less commonly represented data in the model via density weighting.^17^ Approaches to density-weighted regression require choices about the extent of up or down-weighting; in our analysis we wished to assess the relationship between muscle mass and CMI across the biological range of muscle mass, i.e. to balance regression across the range of muscle mass in the cohort. To achieve this, we weighted patient values by the reciprocal of their local Kernel density estimate for the gold standard measure of muscle mass. This resulted in available data across the range of muscle mass having equal contributions to the regression. To avoid the over-influence of extreme outliers the top ten most isolated values were assigned a maximal weighting. In the larger UK Biobank population ~~BIA ALM (normalised to BSA)~~ and HGS was ~~ere~~ available as alternative, less precise, surrogate of muscle mass; similar linear regression and weighted modelling was then explored using CMI as the explanatory variable in this larger sample size.

**14. References**

1. Cruz-Jentoft AJ, Bahat G, Bauer J, et al. Sarcopenia: revised European consensus on definition and diagnosis. *Age Ageing*. Jan 1 2019;48(1):16-31. doi:10.1093/ageing/afy169

2. Du Bois D, Du Bois EF. A formula to estimate the approximate surface area if height and weight be known. 1916. *Nutrition*. Sep-Oct 1989;5(5):303-11; discussion 312-3.

3. Dodds RM, Granic A, Robinson SM, Sayer AA. Sarcopenia, long-term conditions, and multimorbidity: findings from UK Biobank participants. *J Cachexia Sarcopenia Muscle*. Feb 2020;11(1):62-68. doi:10.1002/jcsm.12503

4. Grip Strength Measurement. UK Biobank; 2011.

5. Blood Sample Collection, Processing and Transport. UK Biobank; 2011.

6. Inker LA, Eneanya ND, Coresh J, et al. New Creatinine- and Cystatin C–Based Equations to Estimate GFR without Race. *New England Journal of Medicine*. 2021;385(19):1737-1749. doi:10.1056/nejmoa2102953

7. Inker LA, Schmid CH, Tighiouart H, et al. Estimating glomerular filtration rate from serum creatinine and cystatin C. *N Engl J Med*. Jul 5 2012;367(1):20-9. doi:10.1056/NEJMoa1114248

8. Charlson ME, Pompei P, Ales KL, MacKenzie CR. A new method of classifying prognostic comorbidity in longitudinal studies: development and validation. *J Chronic Dis*. 1987;40(5):373-83. doi:10.1016/0021-9681(87)90171-8

9. Gasparini A. comorbidity: An R package for computing comorbidity scores. *Journal of Open Source Software*. 2018;3(23):648. doi:10.21105/joss.00648

10. Quan H, Sundararajan V, Halfon P, et al. Coding algorithms for defining comorbidities in ICD-9-CM and ICD-10 administrative data. *Med Care*. Nov 2005;43(11):1130-9. doi:10.1097/01.mlr.0000182534.19832.83

11. Quan H, Li B, Couris CM, et al. Updating and Validating the Charlson Comorbidity Index and Score for Risk Adjustment in Hospital Discharge Abstracts Using Data From 6 Countries. *American Journal of Epidemiology*. 2011;173(6):676-682. doi:10.1093/aje/kwq433

12. Gilbert T, Neuburger J, Kraindler J, et al. Development and validation of a Hospital Frailty Risk Score focusing on older people in acute care settings using electronic hospital records: an observational study. *The Lancet*. 2018;391(10132):1775-1782. doi:10.1016/s0140-6736(18)30668-8

13. Williams DM, Jylhävä J, Pedersen NL, Hägg S. A Frailty Index for UK Biobank Participants. *J Gerontol A Biol Sci Med Sci*. Mar 14 2019;74(4):582-587. doi:10.1093/gerona/gly094

14. Fried LP, Tangen CM, Walston J, et al. Frailty in older adults: evidence for a phenotype. *J Gerontol A Biol Sci Med Sci*. Mar 2001;56(3):M146-56. doi:10.1093/gerona/56.3.m146

15. Hanlon P, Nicholl BI, Jani BD, Lee D, McQueenie R, Mair FS. Frailty and pre-frailty in middle-aged and older adults and its association with multimorbidity and mortality: a prospective analysis of 493 737 UK Biobank participants. *Lancet Public Health*. Jul 2018;3(7):e323-e332. doi:10.1016/S2468-2667(18)30091-4

16. Torgo L, Ribeiro RP, Pfahringer B, Branco P. SMOTE for Regression. Springer Berlin Heidelberg; 2013:378-389.

17. Steininger M, Kobs K, Davidson P, Krause A, Hotho A. Density-based weighting for imbalanced regression. *Machine Learning*. 2021/08/01 2021;110(8):2187-2211. doi:10.1007/s10994-021-06023-5
